# Mechanisms of suppression of cell growth by dual inhibition of ALK and MEK in ALK-positive non-small cell lung cancer

**DOI:** 10.1038/s41598-019-55376-4

**Published:** 2019-12-11

**Authors:** N. Shrestha, M. Nimick, P. Dass, R. J. Rosengren, J. C. Ashton

**Affiliations:** Department of Pharmacology & Toxicology, School of Biomedical Sciences, University of Otago, Dunedin, New Zealand

**Keywords:** Drug development, Cancer therapy, Non-small-cell lung cancer

## Abstract

Anaplastic lymphoma kinase (ALK) rearrangement, a key oncogenic driver in a small subset of non-small cell lung cancers, confers sensitivity to ALK tyrosine kinase inhibitors (TKIs). Crizotinib, a first generation ALK-TKI, has superiority to standard chemotherapy with longer progression-free survival and higher objective response rate. However, clinical benefit is limited by development of resistance, typically within a year of therapy. In this study the combined effect of crizotinib and the MEK inhibitor selumetinib was investigated in both crizotinib naïve (H3122) and crizotinib resistant (CR-H3122) ALK-positive lung cancer cells. Results showed that combination treatment potently inhibited the growth of both H3122 and CR-H3122 cells, resulting from increased apoptosis and decreased cell proliferation as a consequence of suppressed downstream RAS/MAPK signalling. The drug combination also elicited a greater than 3-fold increase in Bim, a mediator of apoptosis, and p27, a cyclin dependent kinase inhibitor compared to crizotinib alone. The results support the hypothesis that combining MEK inhibitors with ALK inhibitor can overcome ALK inhibitor resistance, and identifies Bim, PARP and CDK1 as druggable targets for possible triple drug therapy.

## Introduction

Lung cancer is the leading cause of cancer mortality worldwide, accounting for approximately 1.59 million deaths annually^1^. Anaplastic lymphoma kinase (ALK) rearrangements occur in approximately 2% to 7% of non-small cell lung cancer (NSCLC) patients and are more common among younger aged patients with no or light smoking history^2–6^. The EML4-ALK fusion oncogene is the most common ALK rearrangement and was first reported in 2007^4,7^. A chromosomal rearrangement leads to the fusion of a portion of ALK gene and echinoderm microtubule-associated protein-like 4 (EML4) resulting in a constitutively active ALK and aberrant downstream signalling^4,8^.

Crizotinib is a potent, ATP competitive, multi-targeted tyrosine kinase inhibitor of ALK, MET and ROS1^9,10^. Based on remarkable results from early phase studies, crizotinib was approved by the U.S. Food and Drug Administration (FDA) and European Medicines Agency (EMA) for the treatment of ALK rearrangement in NSCLC^11–14^. Following this, the PROFILE 1014 study conclusively demonstrated the superiority of crizotinib compared to standard chemotherapy, with longer progression-free survival (PFS) and a higher objective response rate of 10.9 vs. 7 months and 74% vs. 45%, respectively^15^. Crizotinib has been rapidly followed by second and third generation ALK inhibitors such as ceritinib, alectinib, brigatinib and lorlatinib^16^. However, despite the antitumor activity of crizotinib and other ALK inhibitors, cancer drug resistance develops typically within a few years of treatment.

Mechanisms of resistance to crizotinib involve the alteration of the target gene itself either by mutation or amplification, and activation of bypass signalling pathways. Some mediators in these signalling pathways are druggable targets and have been under investigation for combination drug treatment. Up front combination drug treatment of several targets has been argued as one strategy to delay or overcome drug resistance. Bozic *et al*.^17,18^ have argued that the probability of a combination being curative increases as the frequency of locations in the genome that confer resistance to the combination as a whole if mutated decreases. Following this, we term drug targets “independent” if mutations that provide resistance to drugs against one target in a combination do not necessarily cause resistance to drugs against the other target(s). ALK overexpression and constitutive activation is unique to ALK-positive NSCLC cells; thus, with a highly specific first target established, the search for secondary targets is facilitated.

One strategy to this search is to investigate mediators of bypass signalling pathways as co-targets for combination with ALK inhibitors. These include receptor tyrosine kinases (RTKs) such as epidermal growth factor receptor (EGFR), insulin like growth factor receptor (IGFR), human epidermal growth factor receptor 2 (HER2) and cKIT, or mutation in EGFR or kirsten rat sarcoma (KRAS). These can regulate downstream signalling independently, promoting the growth and survival of cells irrespective of ALK inhibition^19–25^. Promisingly, Hrustanovic *et al*.^26^ have demonstrated *in vitro* and *in vivo* that targeting MEK together with ALK in cancer cells harbouring EML4-ALK is highly effective at supressing cell growth compared to inhibition of either target alone. Up front combination of ALK and MEK inhibition has improved the response in a preclinical model of EML4-ALK NSCLC, and in a patient derived acquired resistance cellular model of EML4-ALK^26,27^.

In this study we further investigated dual inhibition of ALK and MEK in ELM4-ALK cells. We aimed to test the hypothesis that combination ALK/MEK inhibition is consistent with independent drug action as described above. We therefore (i.) tested whether the development of ALK inhibitor resistance lead to cross-resistance to MEK inhibition, and (ii.) tested whether combined drug action was greater than that predicted by a model that assumes a common target (the Loewe model^28^). Finally, we interrogated the pathways by which ALK/MEK inhibition suppressed cancer cell growth so as to identify more druggable targets, as the approach of Bozic *et al*. requires a combination of three drugs or more to maximise suppression of cancer cell growth and prevention of drug resistance.

We used crizotinib, a first-in-class ALK inhibitor, and selumetinib, a potent, non-ATP competitive inhibitor of marker extraction kernel 1/2 (MEK1/2) which inhibits the phosphorylation of MEK resulting in downregulation of RAS/MAPK signalling^29^. We chose selumetinib because it has demonstrated potent anti-tumour activity in preclinical and clinical trials of various cancers including NSCLC^30–32^. We investigated the combined effect of crizotinib with selumetinib in both crizotinib naïve and crizotinib resistant ALK-positive lung cancer cells. We confirmed that the combination caused a greater reduction of cell viability compared to single drug treatments, and that this effect was consistent with independent drug action. We also observed, a significant decrease in cell proliferation via G1 arrest and collapse of the S phase, and induction of apoptosis. This led us to determine key roles for Bim, PARP and CDK1, all of which are druggable targets. Our findings therefore add support to the clinical investigation of dual ALK/MEK inhibition therapy as a strategy to delay or overcome drug resistance in ALK-positive lung cancer, and points the way toward possible drug therapies with three or more targets.

## Methods and Materials

### Materials

Crizotinib and selumetinib were purchased from LC laboratories (Woburn, Massachusetts, USA). Bovine serum albumin (BSA), Foetal bovine serum (FBS), Rosswell park memorial institute medium (RPMI), penicillin/streptomycin were purchased from Life Technologies (Auckland, New Zealand). Precision plus protein kaleidoscope, acrylamide (1:30) were obtained from Bio-Rad Laboratories (Hercules CA, USA). CL-XPosure film, supersignal west pico were obtained from Thermofisher (Auckland, New Zealand). Propidium iodide was purchased from Sigma- Aldrich (St louis, MO, USA). FxCycle PI/RNase was from Life technologies (California, USA). Annexin V-APC and Ac-DEVD-AFC caspase-3 fluorogenic substrate was purchased from BD Biosciences (New Jersey, USA).

Antibodies against ALK(D5F3), phosphorylated-ALK (Tyr1604), Phospho-p44/42 MAPK (Erk1/2) (Thr202/Tyr204), Bim, Bcl2, caspase, cleaved caspase, PARP, cleaved PARP, cyclinD1, p27 were purchased from Cell Signaling Technology (Danvers, MA, USA). Erk1/2 and β-tubulin antibodies were purchased from Sigma-Aldrich (St louis, MO, USA). HRP-conjugated goat anti-rabbit and HRP-conjugated goat anti-mouse were obtained from Calbiochem (San Diego, CA, US).

### Cell culture

The human adenocarcinoma ALK-positive non-small cell lung cancer (H3122) cell line harbouring EML4-ALK variant 1 fusion gene was gifted from Professor Daniel Costa, Harvard University. We used this cell line as it contains the most common ELM4-ALK variant (1) which also has good sensitivity to ALK inhibitors^33,34^. Human adenocarcinoma non-small cell lung cancer (A549) cell line harbouring K-RAS gene codon 12-point mutation were used as a non-ALK control, and were kindly provided by Dr Gregory Giles, University of Otago. Crizotinib-resistant (CR-H3122) cells were generated as described in Wilson *et al*.^35^ and were maintained in 0.8 µM of crizotinib. Briefly, H3122 cells were cultured with increasing concentrations of crizotinib starting from 0.4 µM for 24 h followed by 0.56 µM for next 24 h. Cells were then maintained in 0.80 µM from 3^rd^ day to 4 months. Media was changed every 2–3 days supplemented with fresh drug.

All cells lines were maintained in RPMI medium supplemented with 100 U/ml of penicillin, 100 μg/ml of streptomycin and 10% (CR-H3122), 5% (H3122), 2% (A549) of fetal bovine serum (FBS). Cells were incubated at 37 °C, 5% CO2, 95% humidified air.

### Cell viability assay

H3122, A549 and CR-H3122 were seeded into 96 wells plate at a density of 7 × 10^3^, 4 × 10^3^ and 10 × 10^3^ cells per well respectively and incubated for 24 h. Next, cells were treated with indicated concentration of drug and further incubated for 72 h followed by fixation with trichloroacetic acid (TCA). Cell viability was determined using the sulforhodamine B (SRB) assay, as described in Vichai *et al*.^36^. The concentration of each drug required to reduce the cell viability by 50% (IC_50_) was determined by nonlinear regression using Graphpad Prism software, from three independent experiments performed in triplicate.

### Cell cycle assay

H3122 (3 × 10^5^ cells/well) and CR-H3122 (3.5 × 10^5^ cells/well) cells were seeded in 6-well culture plates and incubated for 24 h prior to drug treatment. H3122 cells were treated with indicated concentration of vehicle (DMSO 0.1%), crizotinib (0.25 µM), selumetinib (7.5 µM) and their combination. CR-H3122 cells were treated with vehicle (DMSO 0.1%), crizotinib (2.5 µM), selumetinib (7 µM) and their combination. Cells were then incubated for 24 and 48 h. At the end of treatment period, cells were washed with isotonic PBS, collected by trypsinisation and centrifuged. The cell pellets were then fixed with 70% ethanol. Next, cells were washed with ice-cold 0.01 M PBS and centrifuged. Finally, the cell pellet was resuspended in FxCycle PI/RNase staining solution and incubated in the dark for 30 min at room temperature. The samples were then analysed by Gallious BD flow cytometer and data obtained were analysed with FlowJo LLC software. Three independent experiments were performed in triplicate and the results are expressed as cell number in each phase, as a percent of total cell number.

### Apoptosis assay

H3122 (3 × 10^5^ cells/well) and CR-H3122 (3.5 × 10^5^ cells/well) cells were seeded in 6-well culture plates and incubated for 24 h prior to drug treatment. H3122 cells were treated with vehicle (DMSO 0.1%), crizotinib (0.25 µM), selumetinib (7.5 µM) and their combination. and incubated for 24, 48 and 72 h. CR-H3122 cells were treated with vehicle (DMSO 0.1%), crizotinib (2.5 µM), selumetinib (7 µM) and their combination followed by incubation for 24 and 48 h. Cells were then washed with 0.01 M PBS, collected by trypsinisation and centrifuged. The cell pellet was resuspended in 100 µl of binding buffer (10 mM HEPES, 140 mM NaCl, 2.5 mM CaCl2). Annexin V-APC, propidium iodide (50 µg/ml) and 200 µl of binding solution was added to each sample and incubated in the dark at room temperature. Finally, the samples were analysed on Gallious BD flow cytometer. Data obtained were analysed using Kaluza analysis Software. Three independent experiments were performed in triplicate and the results are expressed as the number of apoptotic cells as a percent of total cell number.

### Western blotting

H3122 and CR-H3122 cells were seeded in petri dishes at the density of 2.0 × 10^6^ per dish and incubated for 24 h. H3122 cells were treated with indicated concentration of vehicle (DMSO 0.1%), crizotinib (0.25 µM), selumetinib (7.5 µM) and their combination. CR-H3122 cells were treated with vehicle (DMSO 0.1%), crizotinib (2.5 µM), selumetinib (7 µM) and their combination. Cells were then incubated for 24 h. Cells were lysed with lysis buffer (50 mM Tris base (pH-7.5), 150 mM NaCl, 1 mM EDTA, 1 mM EGTA, 0.5% NP-40, 0.5% SDS, 1 mM sodium orthovanadate, 2.5 mM sodium pyrophosphate, 10 mM sodium fluoride, 1 mM PMSF and complete protease and phosphatase inhibitor cocktail). Cells were then collected, sonicated and centrifuged at 14000 rpm for 10 min at 4 °C. 10 µg of protein was subjected to SDS-PAGE and analysed by Western blotting.

### Caspase assay

H3122 (3 × 10^5^ cells/well) and CR-H3122 (3.5 × 10^5^ cells/well) cells were seeded in 6-well culture plates and incubated for 24 h. H3122 cells were treated with vehicle (DMSO 0.1%), crizotinib (0.25 µM), selumetinib (7.5 µM) and their combination for 48 h. CR-H3122 cells were treated with vehicle (DMSO 0.1%), crizotinib (2.5 µM), selumetinib (7 µM) and their combination for 24 h. H3122 and CRH3122 cells were treated with 5 µM and 100 µM of cisplatin (positive control), respectively. Cells were then collected, centrifuged and washed with 0.01 M PBS. Supernatant was discarded and cell pellets were suspended in 100 µl buffer (100 mM HEPES pH 7.5, 10% sucrose, 0.1% CHAPS and 0.0001%NP-40) supplemented with DTT and caspase substrate and further incubated for 30 min at 37 °C. The samples were diluted to 1:5 in buffer (100 mM HEPES pH 7.5, 10% sucrose, 0.1% CHAPS and 0.0001%NP-40) and examined using a spectrofluorometer with excitation and emission wavelengths of 380 and 500 nm, respectively.

### Data analysis

Cell viability data were normalised to control and analysed by nonlinear regression model using Graphpad Prism 7 software. Cell cycle and apoptosis data were analysed using a two-way ANOVA using time as one factor and drug treatment as the other, coupled with a Bonferroni post-hoc test. All other data that did not involve time were analysed using a one-way ANOVA coupled with a Bonferroni post-hoc test. Data are presented as the mean ± SEM. P < 0.05 was the minimal requirement for a statistically significant difference.

To determine if crizotinib and selumetinib suppress cell growth in a manner consistent with independent drug action in both H3122 and CR-H3122 cells, we modelled combination treatment data by calculating the combination index (CI) described by Chou and Talalay^37^ using CompuSyn software. Commonly used in the study of drug synergy (positive interaction), this approach is derived from the zero-interaction (additivity) model of Loewe. This model is based on the assumption of a shared target for two drugs. Therefore, an effect greater than predicted by Loewe additivity (Chou-Talaly CI < 1) means that central assumption of the Loewe model is falsified; thus, that the drugs act on separate drug targets. Determining whether drugs that act on separate targets have a positive interaction (synergy) requires a different zero-interaction model (the Bliss model)^38^. We did not carry out this analysis, as testing for independent drug action rather than synergy was our aim.

## Results

### Crizotinib in combination with selumetinib suppression of the growth of ALK-positive NSCLC cells

**Figure 1:**
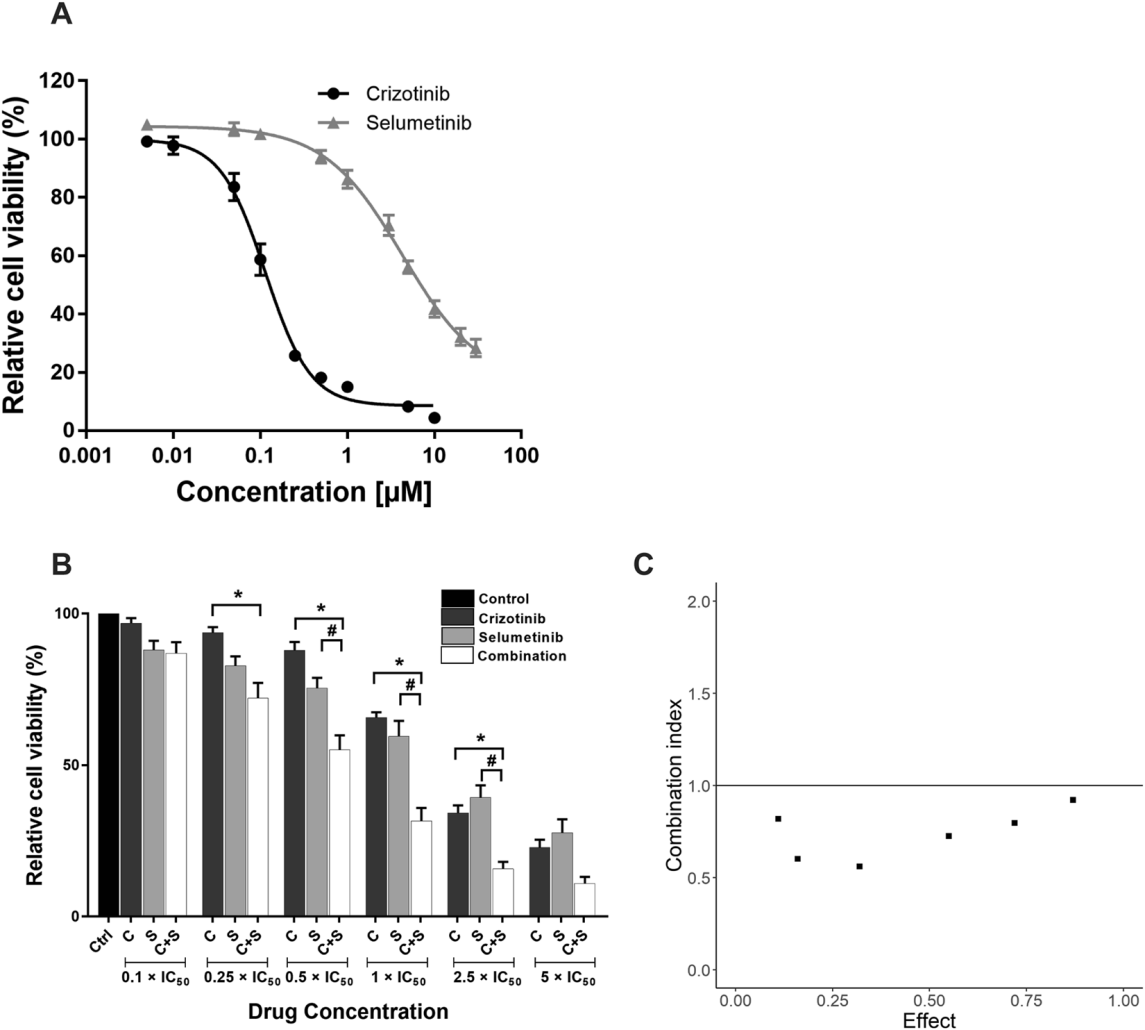
Effect of crizotinib, selumetinib and their combination on crizotinib naïve H3122 cells. (**A**) Effects on cell viability by the ALK inhibitor; crizotinib and MEK inhibitor; selumetinib, data points are means and error bars are SEM. (**B**) Cell viability of a combination of crizotinib and selumetinib. Bar denotes; ■ DMSO control, ■ crizotinib,  selumetinib and □ combination of both. Errors bars are SEM. Significance was determined using Bonferroni post-hoc tests following one-way-ANOVA. (**C**) Combination index plot for drug combination of crizotinib and selumetinib. The horizontal line represents Loewe additivity. All data represent three independent experiments in triplicate. *p < 0.05 for crizotinib vs. combination and ^#^p < 0.05 for selumetinib vs. combination.

The relative potency of crizotinib and selumetinib was initially examined in ALK-positive (H3122) and ALK-negative (A549) non-small cell lung cancer cells. H3122 cells were highly sensitive to crizotinib compared to selumetinib, with cell viability IC_50_ values of 0.1 and 3 µM, respectively (Fig. 1A). Sensitivity to crizotinib by ALK-negative A549 cells was markedly less than for ALK-positive cells, (IC_50_ of 0.8 µM). IC_50_ values for selumetinib were similar in A549 cells as in H1322 cells (2 µM) (Fig. S1A). The effect of crizotinib and selumetinib in combination was then examined in H3122 and A549 cells. Six different combinations were selected using equivalent potencies relative to each drug’s IC_50_ value. For each concentration, the two drugs alone were compared with the combination of the two. Chou-Talalay analysis was performed to determine if the two drugs acted greater than Loewe additivity^37^. In H3122 cells, 3 of the 6 drug combinations examined significantly reduced cell viability compared to both single drug treatments (p < 0.05) (Fig. 1B) and all drug combinations showed greater than Loewe additivity (Chou-Talalay Combination index < 1) (Fig. 1C). By contrast, the combined effect of crizotinib and selumetinib was solely driven by selumetinib in A549 cells (Supplementary Fig. S1B). Furthermore, combination indices showed that all combinations except the higher concentration of the drugs were mutually antagonistic (CI > 1) (Supplementary Fig. S1C).

### Effect of crizotinib and selumetinib combination treatment on downstream signalling pathway and cell cycle mediators

**Figure 2:**
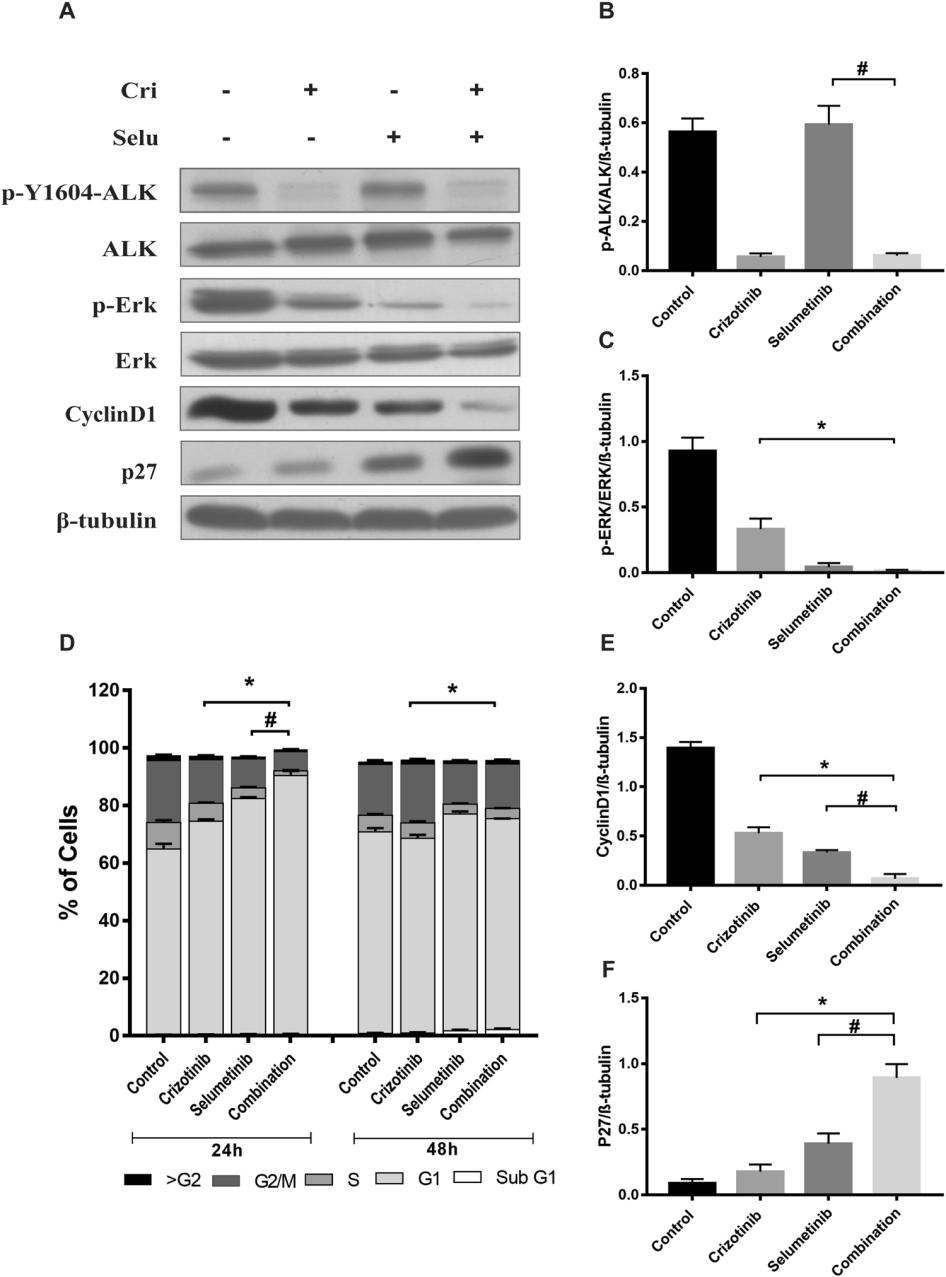
Effect of combination treatment on cell cycle progression and downstream RAS/MAPK signalling pathway in H3122 cells. (**A**) Representative Western blots of ALK, pALK, ERK, p-ERK, cyclinD1, p27 after 24 h exposure of drugs treatment. Densitometry of Western blots of (**B**) pALK/ALK (**C**) pERK/ERK. Significance was determined using Bonferroni post-hoc tests following one-way-ANOVA. (**D**) Cell cycle distribution after treatment with indicated concentration of crizotinib, selumetinib and their combination for 24 and 48 h. Bar denotes; ■ > G2, ■ G2/M,  S,  G1, □ sub G1. Significance was determined by Bonferroni post-hoc tests following two-way-ANOVA. Densitometry of Western blots of (**E**) CyclinD1 (**F**) p27. Significance was determined by one-way-ANOVA with Bonferroni post-hoc test. All data are presented as mean ± SEM. Three independent experiment were performed. *p < 0.05 for crizotinib vs. combination and ^#^p < 0.05 for selumetinib vs. combination.

To investigate the mechanisms involved in suppression of cell growth by the combination treatment, key proteins downstream of the RAS/MAPK signalling pathway were examined. Western blotting was performed to determine the expression and activation of ALK and MEK, by measuring ALK, phosphorylated-ALK (pALK), ERK and phosphorylated-ERK (pERK). After 24 hours, pALK/ALK was decreased 90% by both crizotinib alone and the combination of crizotinib and selumetinib, whereas there was no change following selumetinib (Fig. 2B). Additionally, pERK/ERK was reduced 64% by crizotinib, 95% by selumetinib, and 99% by the drugs in combination (p < 0.05) (Fig. 2C).

The impact of combination treatment on cell cycle progression was then investigated. Cell cycle analysis showed that both crizotinib and selumetinib as single drug treatments promoted the accumulation of H3122 cells in the G1 phase of the cell cycle. Combination of the two drugs increased the percentage of cells in the G1 phase even further. This was accompanied by suppression of the number of S phase cells by greater than 77% compared to either of the single drug treatments (p < 0.05) (Fig. 2D). Therefore, the expression of proteins involved in the G1 to S phase transition such as cyclin D1 and the cyclin dependent kinase inhibitor, p27, were examined using Western blotting. Combination drug treatment significantly decreased the protein expression of cyclin D1 (>80%) compared to single drug treatments (Fig. 2E). Moreover, the expression level of p27 was markedly increased by 4- and 1.3-fold by the combination treatment compared to crizotinib or selumetinib alone (p < 0.05) (Fig. 2F). Hence, selumetinib enhanced the anti-proliferative effect of crizotinib by increasing G1 phase arrest, which resulted from a decrease in cyclin D1 and an increase in p27 expression.

### Crizotinib and selumetinib combine to induce mediators of apoptosis

**Figure 3:**
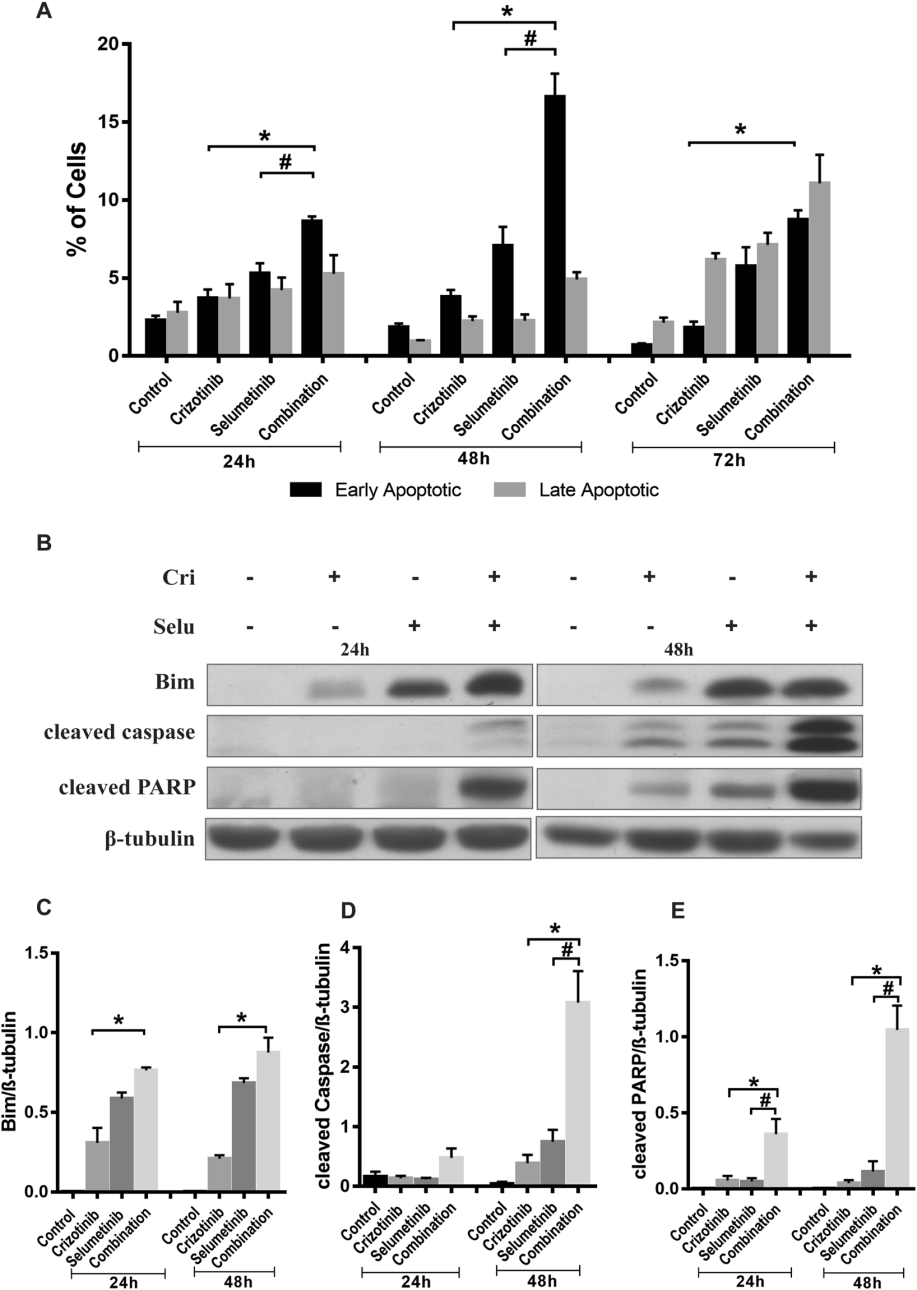
Effect of combination treatment on induction of apoptosis in H3122 cells. (**A**) Percentage of apoptotic cells after treatment with indicated concentration of crizotinib, selumetinib and their combination for 24, 48, and 72 h. Bar denotes; ■ Early apoptotic,  late apoptotic Significance was determined using a two-way-ANOVA coupled with a Bonferroni post-hoc test. (**B**) Representative Western blots of apoptotic marker after 24 and 48 h exposure of drugs treatment. Densitometry of Western blots of (**C**) Bim (**D**) cleaved Caspase (**E**) cleaved PARP. Significance was determined by Bonferroni post-hoc tests following one-way-ANOVA. All data are presented as mean ± SEM. Three independent experiment were performed. *p < 0.05 for crizotinib vs. combination and ^#^p < 0.05 for selumetinib vs. combination.

Crizotinib also exhibits anti-tumour activity via induction of apoptosis (Christensen *et al*., 2007). Similarly, selumetinib, a potent MEK1/2 inhibitor, downregulates the RAS/MAPK signalling pathway leading to activation of downstream apoptotic signalling^39^. Therefore, whether the combination of crizotinib with selumetinib could enhance induction of apoptosis in H3122 cells was investigated. Following drug treatment, apoptosis was initiated at 24 h, reached maximum at 48 h and entered late apoptotic phase at 72 h (Fig. 3A). After 48 h, the percentage of apoptotic H3122 cells was only slightly increased by crizotinib or selumetinib treatment (4 or 7%, respectively), whereas the combination treatment resulted in significant increase in the percentage of apoptotic cells (17%) (p < 0.05). To determine the drivers of apoptosis, the expression of key regulators of intrinsic apoptosis Bim, Bcl2, cleaved caspase, and cleaved PARP were examined by Western blotting. Combination treatment significantly increased the endogenous expression of Bim by 3 and 0.3-fold, respectively compared to crizotinib and selumetinib alone (p < 0.05). However, there was no change in Bcl2 expression (Supplementary Fig. S2B). After 24 h of drug treatment, cleaved caspase expression was negligible but was significantly increased after 48 h of combination treatment (Fig. 3D). This observation is consistent with results from apoptosis assay suggesting that induction of apoptosis was maximal after 48 h of treatment. Combination treatment significantly increased cleaved caspase expression by 3 to 7-fold compared to either drug alone. To confirm this, we carried out a caspase assay, and found sharp increase in caspase activity with combination treatment consistent with result from Western blotting (Supplementary Fig. S2C). Expression of cleaved PARP was markedly increased by 8 to 26-fold from combination compared to either drug alone (p < 0.05) (Fig. 3E). These results show that the combination of crizotinib and selumetinib significantly increased apoptosis by increasing the expression of Bim, cleaved caspase and cleaved PARP to a degree that not reached by crizotinib alone.

### Effect of combination of crizotinib and selumetinib in crizotinib resistant ALK-positive NSCLC cells

**Figure 4:**
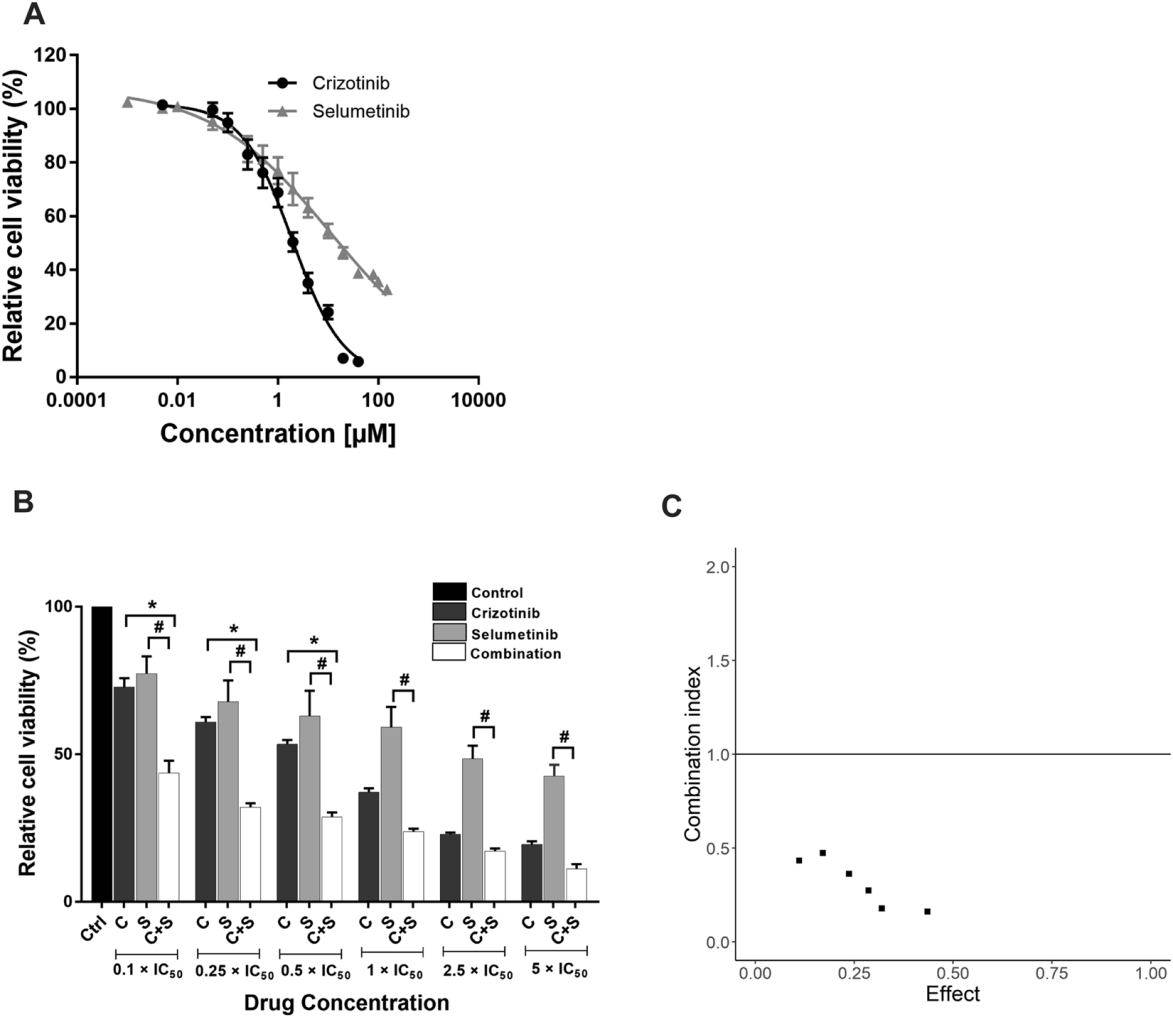
Crizotinib combines with selumetinib to suppress growth of crizotinib resistance CRH3122 cells. (**A**) Effects on cell viability by crizotinib and selumetinib, data points are means and error bars are SEM. (**B**) Effect on cell viability by a combination of crizotinib and selumetinib. Bar denotes; ■ DMSO control, ■ crizotinib,  selumetinib and □ combination of both. Errors bars are SEM. Significance was determined using Bonferroni post-hoc tests following one-way-ANOVA. (**C**) Combination index plot for drug combination of crizotinib and selumetinib. The horizontal line represents Loewe additivity. All data represent three independent experiments in triplicate. *p < 0.05 for crizotinib vs. combination and ^#^p < 0.05 for selumetinib vs. combination.

The anti-proliferative effect of crizotinib or selumetinib and their combination was then examined in crizotinib resistant ALK-positive NSCLC cells (CR-H3122)^35^. Sensitivity of CR-H3122 cells to crizotinib was highly reduced compared to H3122 cells, with an IC_50_ of 2.3 µM (22-fold over parental H3122 cells). The IC_50_ for selumetinib was elevated in CR-H1322 cells to 11 µM, a 3-fold increase compared to H3122 (Fig. 4A). Results from combination drug treatment showed that all six combinations significantly supressed the growth of CR-H3122 cells compared to single drug treatment (p < 0.05) (Fig. 4B). Interestingly, combination crizotinib with selumetinib was highly potent in the resistant cells compared to the parental H3122 cells (Chou-Talaly Combination Indices < 0.3) (Fig. 4C).

**Figure 5:**
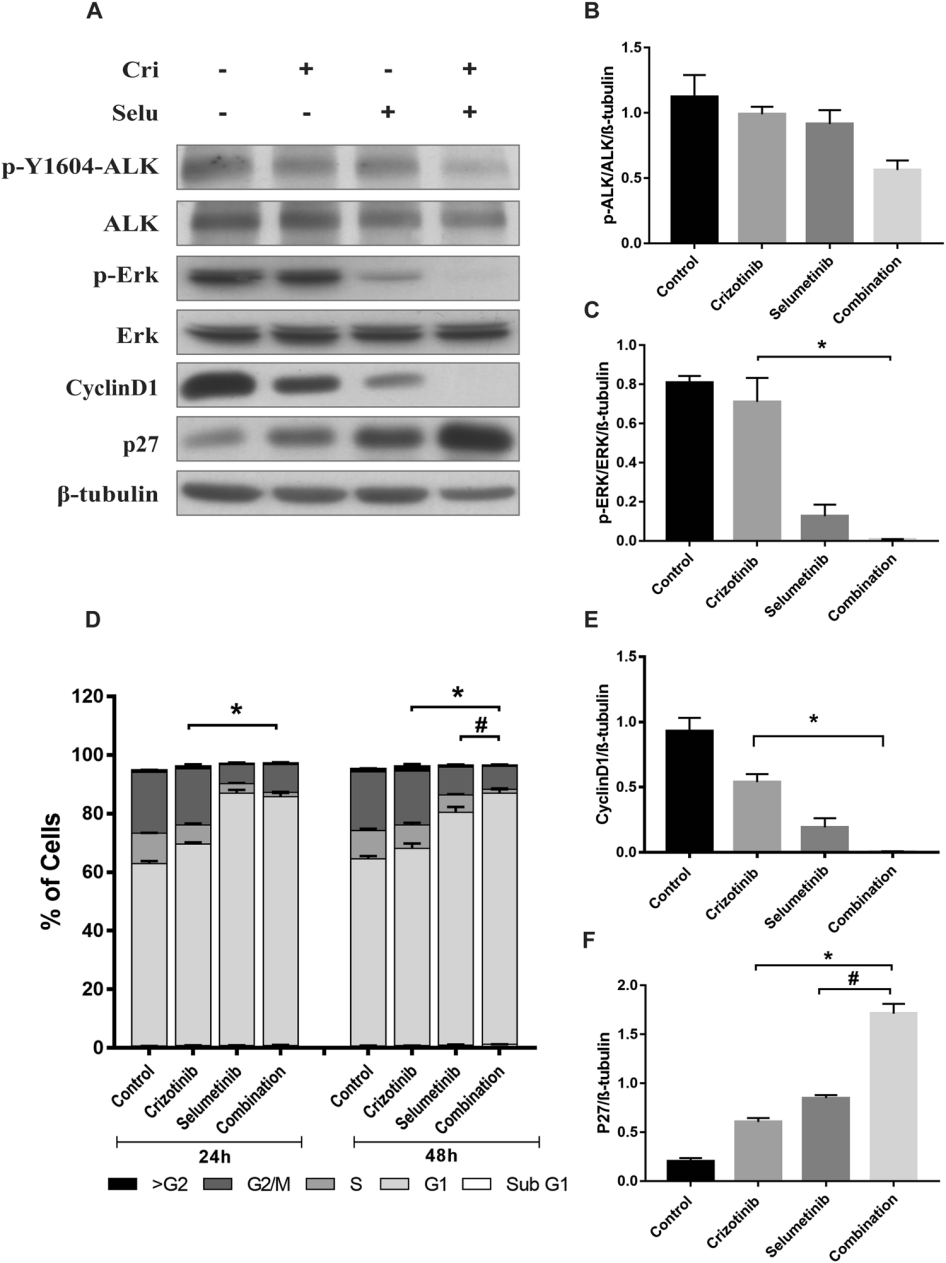
Combination crizotinib/selumetinib treatment induces G1 phase arrest and inhibist pERK expression in CRH3122 cells. (**A**) Representative Western blots of ALK, pALK, ERK, p-ERK, cyclinD1, p27 after 24 h exposure of drugs treatment. Densitometry of Western blots of (**B**) pALK/ALK (**C**) pERK/ERK. Significance was determined using Bonferroni post-hoc tests following one-way-ANOVA. (**D**) Cell cycle distribution after treatment with indicated concentration of crizotinib, selumetinib and their combination for 24 and 48 h. Bar denotes; ■ > G2, ■ G2/M,  S,  G1, □ sub G1. Significance was determined using Bonferroni post-hoc tests to compare combination and single drug treatments following a two-way-ANOVA using time and drug treatment as factors. Densitometry of Western blots of (**E**) CyclinD1 (**F**) p27. Significance was determined using Bonferroni post-hoc tests following one-way-ANOVA. All data are presented as mean ± SEM. Three independent experiment were performed. *p < 0.05 for crizotinib vs. combination and ^#^p < 0.05 for selumetinib vs. combination.

To further evaluate the mechanisms for the drug combination effects on crizotinib resistant cells, proteins downstream of the RAS/MAPK signalling pathway, cell cycle progression and apoptosis were all examined. Crizotinib alone had almost no effect on the phosphorylation of ALK and ERK in CR-H1322 cells, consistent with ALK inhibitor resistance. Nor was there any significant reduction of ALK phosphorylation by combination of crizotinib with selumetinib (Fig. 5B). However, the cells remained sensitive to ERK phosphorylation by selumetinib, with a 93% decrease in pERK/ERK protein expression after 24 h. Moreover, the combination of crizotinib and selumetinib decreased the protein expression of pERK/ERK by 99% (p < 0.05).

**Figure 6:**
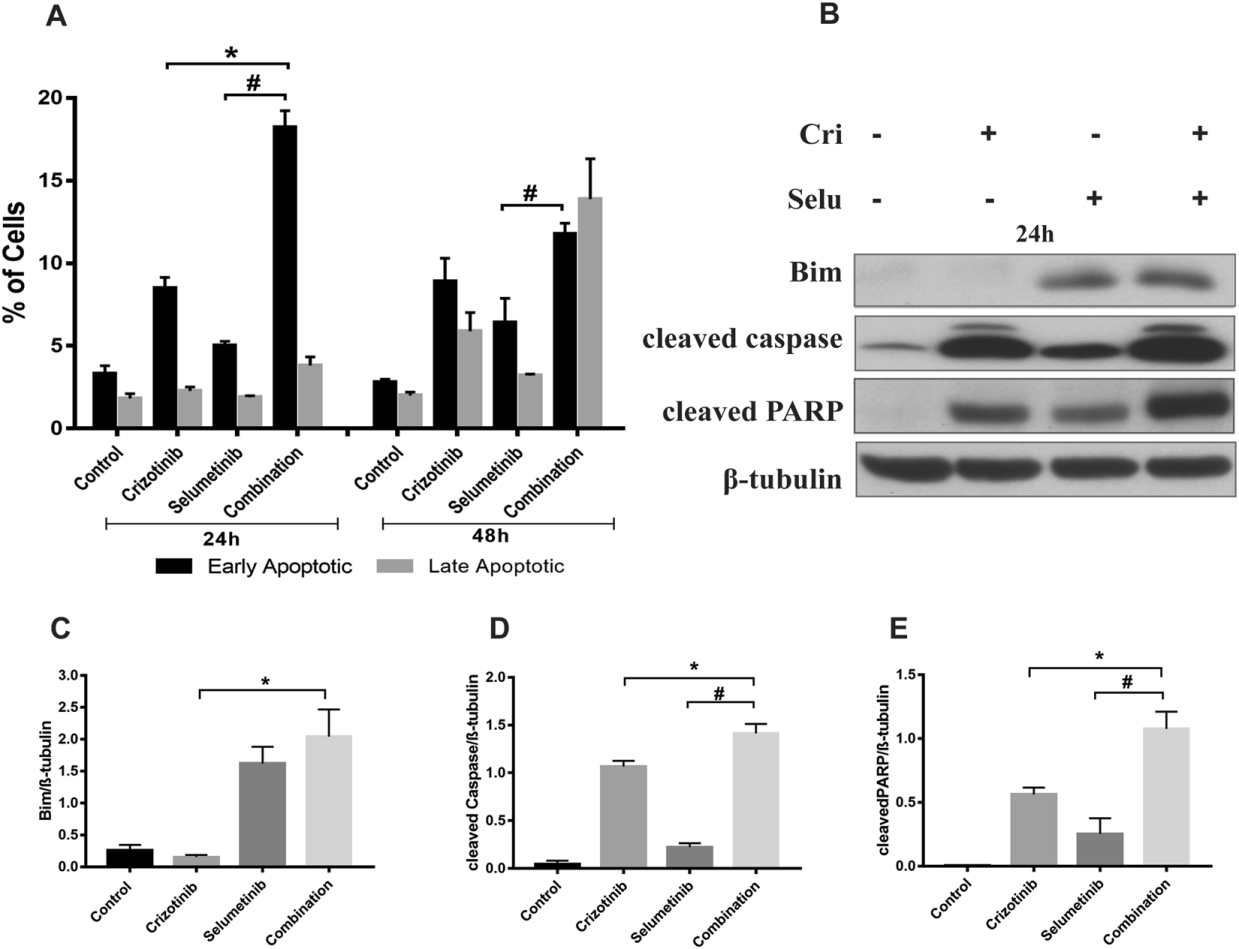
Treatment with crizotinib and selumetinib induced apoptosis in CRH3122 cells. (**A**) Percentage of apoptotic cells after treatment with indicated concentration of crizotinib, selumetinib and their combination for 24 and 48 h. Bar denotes; ■ Early apoptotic,  late apoptotic. Significance was determined using Bonferroni post-hoc tests following two-way-ANOVA. (**B**) Representative Western blots of apoptotic marker after 24 and 48 h of drugs treatment. Densitometry of Western blots of (**C**) Bim (**D**) cleaved Caspase (**E**) cleaved PARP. Significance was determined using Bonferroni post-hoc tests to compare combination and single drug treatments following a two-way-ANOVA using time and drug treatment as factors. All data are presented as mean ± SEM. Three independent experiment were performed. *p < 0.05 for crizotinib vs. combination and ^#^p < 0.05 for selumetinib vs. combination.

Cell cycle analysis showed that after 24 and 48 h the combination treatment significantly increased the percentage of cells arrested in the G1 phase compared to crizotinib (p < 0.05) (Fig. 5A). Similarly, apoptosis assay showed that combination treatment significantly increased the percentage of early apoptotic cells (18%) compared to crizotinib (8%) or selumetinib (5%) after 24 h (p < 0.05) (Fig. 6A). Lastly, Western blotting was performed to determine the expression of cyclin D1, p27, Bim, Bcl2, cleaved caspase and cleaved PARP. Combination treatment significantly increased the expression of p27, Bim, cleaved caspase and cleaved PARP by 1.83, 12, 0.3, 0.9-fold, respectively, whereas, cyclin D1 was decreased by 1-fold. Similar to what was observed in H3122 cells, there was no change in expression of Bcl2 compared to crizotinib (p < 0.05) (Figs. 5E,F, 6C–E and S3C)

## Discussion

The identification of an ALK-rearrangement oncogene as a therapeutic target and the development of potent ALK tyrosine kinase inhibitors (TKIs) has revolutionised treatment for ALK-positive patients^40^. But despite initial responses, complete and lasting response to ALK-TKI monotherapy is rare to non-existent, as patients typically relapse within a few years at most^41^. Overcoming ALK inhibitor resistance is therefore an area of major research effort. Combination therapy targeting primary oncoproteins and their critical effectors can be an effective alternative therapy that can overcome acquired resistance and improve therapeutic efficacy. The MAPK pathway has been identified as one such key bypass mediator for ALK-positive NSCLC^26^. Building on the work of Hrustanovic *et al*.^26^ we focused on the MAPK pathway; targetting MEK, which is an early mediator upstream of ERK but downstream of ALK and other RTKs. We further found that the combination of crizotinib and selumetinib inhibited cell viability in H3122 cells to a greater extent than predicted by Loewe additivity; importantly this property was found to an even greater degree in crizotinib resistant CR-H1322 cells. This supports the use of combination drug treatment over sequential treatment. By contrast, in an earlier study, we found that the combination of an IGF-1R inhibitor with crizotinib was no more effective in our crizotinib resistant cells than switching to the IGF-1R inhibitor alone^35^.

Our results leave open the question of whether to start combination therapy at the beginning of treatment, or only after the development of resistance to crizotinib. Upfront combination theory is supported by the work of Bozic *et al*.^17,18^ who concluded that simultaneous combination treatment can be more effective than sequential therapy in delaying the emergence of mutations or combinations of mutations that confer resistance to both drugs^17^. Thus, an upfront combination of the two drugs could suppress the accumulation of mutations and ultimately extend the time to relapse. However, as we found that the development of crizotinib resistance caused a small but statistically significant cross-resistance to selumetinib in ELM4-ALK-positive cells (IC_50_ values of crizotinib and selumetinib were increased by 22- and 3-fold, respectively, compared to parental H3122 cells), our results do not support the hypothesis that there are no mutations that may cause cross resistance between these two drug targets.

We have not yet determined the precise mechanisms of resistance in CR-H1322 cells. We have previously shown that proteins such as SRC are upregulated in these cells, and that constitutive phosphorylation of ALK is also upregulated^35^. We have now sequenced the ALK kinase domains in both H3122 and CR-H3122 cells and have found no differences between them (Supplementary Tables 1 and 2). We hypothesise that ALK activation is dysregulated or ALK copy number is increased in CR-H3122 cells, and are currently carrying out whole genome analysis to investigate these hypotheses.

Our approach adds to the findings of Hrustanovic *et al*.^26^ who found that combination ALK/MEK inhibition prevented the emergence of resistance in preclinical models of ALK-positive NSCLC^26^. The authors show that is because of re-activation of the RAS/MAPK pathway via diverse mechanisms of resistance (re-activation of ALK as well as activation of oncogenes such as EGFR, IGFR, HER2, cKIT)^19–26^. Our investigations of the signalling pathways underlying mechanisms showed that the drug combination leads to a strong downregulation of the RAS/MAPK signalling pathway that in turn promotes both cell cycle arrest and apoptosis. In H3122 and CR-H3122 cells, combination treatment significantly decreased the protein phosphorylation of pERK/ERK compared to crizotinib as a single drug treatment. Both crizotinib and selumetinib are known to exhibit anti-tumour activity via G1 cell cycle arrest and induction of apoptosis in various cancers including NSCLC^42–44^ and their combination further increased this G1 phase arrest and suppression of the S phase. Further experiments showed that this is in part mediated by decrease in the expression of cyclin D1 and an increase in the expression of p27. Consistent with this, the RAS/MAPK pathway is involved in the regulation of G1 cell cycle proteins. Phosphorylation of ERK leads to migration to the nucleus and activation of cyclin D1 and downregulation of p27. Thus, ERK plays an important role for entry into the S phase of the cell cycle^45^.

Combination treatment also significantly increased apoptosis compared to individual drugs in both crizotinib naïve and crizotinib resistant ALK-positive NSCLC cells. Specifically, cleaved caspase and cleaved PARP were markedly increased following combination treatment, which was mediated by Bim, a pro-apoptotic protein in the Bcl2 family (though Bcl2 itself was not changed). Both crizotinib and selumetinib individually increased the expression of Bim which is consistent with other studies in B-cell lymphoma and NSCLC cells^46,47^. The mechanism behind the increase in expression of Bim is associated with a decrease in the phosphorylation of ERK, as it is well known that the RAS/MAPK signalling pathway phosphorylates Bim and promotes its proteasomal degradation thereby supressing apoptosis^48^. Similarly, a significant increase in cleaved caspase and cleaved PARP was observed in CR-H3122 cells following combination treatment. This effect was driven mostly by crizotinib where the contribution of selumetinib was minimal. Following selumetinib treatment, apoptosis was a Bim-dependent process, but apoptosis induced by crizotinib was independent of Bim. This may be because crizotinib can inhibit the RAS/MAPK pathway along with other ALK downstream pathways such as AKT/mTOR or JAK/STAT that may contribute to apoptosis with involvement of other pro- or anti-apoptotic proteins^49,50^. Future studies will be required to determine mechanism(s) involved in crizotinib resistant cell lines.

Notably, Bim and CDK1 mediate complementary aspects of tumour cell suppression, namely cell death and proliferation, respectively. Both are druggable targets, Bim by BH3 analogues, and CDK1 by specific inhibitors. Hence our results suggest that triple or even quadruple drug treatments should be screened (i.e., ALK, MEK, Bim, and PARP targeting) in the quest for even greater and longer-term cancer suppression.

## Supplementary information

Supplementary material

## Data Availability

Raw data is available on request.

## References

[1] Ferlay J (2015). Cancer incidence and mortality worldwide: sources, methods and major patterns in GLOBOCAN 2012. Int J Cancer.

[2] Wong DW (2009). The EML4-ALK fusion gene is involved in various histologic types of lung cancers from nonsmokers with wild-type EGFR and KRAS. Cancer.

[3] Koivunen JP (2008). EML4-ALK fusion gene and efficacy of an ALK kinase inhibitor in lung cancer. Clin. Cancer Res..

[4] Soda M (2007). Identification of the transforming EML4–ALK fusion gene in non-small-cell lung cancer. Nature.

[5] Perner S (2008). EML4-ALK fusion lung cancer: a rare acquired event. Neoplasia.

[6] Shaw AT (2009). Clinical features and outcome of patients with non-small-cell lung cancer who harbor EML4-ALK. J. Clin.Oncol..

[7] Rikova K (2007). Global survey of phosphotyrosine signaling identifies oncogenic kinases in lung cancer. Cell.

[8] Roskoski R (2013). Anaplastic lymphoma kinase (ALK): Structure, oncogenic activation, and pharmacological inhibition. Pharmacol. Res..

[9] Ou SH (2011). Activity of crizotinib (PF02341066), a dual mesenchymal-epithelial transition (MET) and anaplastic lymphoma kinase (ALK) inhibitor, in a non-small cell lung cancer patient with de novo MET amplification. J. Thorac. Oncol..

[10] Bergethon K (2012). ROS1 rearrangements define a unique molecular class of lung cancers. J. Clin. Oncol..

[11] Kazandjian D (2014). FDA Approval Summary: Crizotinib for the Treatment of Metastatic Non-Small Cell Lung Cancer With Anaplastic Lymphoma Kinase Rearrangements. Oncologist.

[12] Kwak EL (2010). Anaplastic lymphoma kinase inhibition in non–small-cell lung cancer. NEJM.

[13] Shaw AT (2013). Crizotinib versus chemotherapy in advanced ALK-positive lung cancer. NEJM.

[14] Camidge DR (2012). Activity and safety of crizotinib in patients with ALK-positive non-small-cell lung cancer: Updated results from a phase 1 study. Lancet Oncol..

[15] Solomon BJ (2014). First-Line Crizotinib versus Chemotherapy in ALK -Positive Lung Cancer. NEJM.

[16] Lin JJ, Riely GJ, Shaw AT, Targeting ALK (2017). Precision medicine takes on drug resistance. Cancer Discov..

[17] Bozic I (2013). Evolutionary dynamics of cancer in response to targeted combination therapy. Elife.

[18] Bozic A, Nowak AK (2016). Resisting Resistance. Ann. Rev. Cancer Biol..

[19] Tanizaki J (2012). Activation of HER family signaling as a mechanism of acquired resistance to ALK inhibitors in EML4-ALK-positive non-small cell lung cancer. Clin. Cancer Res..

[20] Choi YL (1734). EML4-ALK mutations in lung cancer that confer resistance to ALK inhibitors. NEJM.

[21] Katayama R (2012). Mechanisms of Acquired Crizotinib Resistance in ALK-Rearranged Lung Cancers. Sci. Transl. Med..

[22] Sasaki T (2011). A novel ALK secondary mutation and EGFR signaling cause resistance to ALK kinase inhibitors. Cancer Res..

[23] Doebele RC (2012). Mechanisms of resistance to crizotinib in patients with ALK gene rearranged non-small cell lung cancer. Clin. Cancer Res..

[24] Zhang S (2011). Crizotinib-resistant mutants of EML4-ALK identified through an accelerated mutagenesis screen. Chem. Biol. Drug Des..

[25] Heuckmann JM (2011). ALK mutations conferring differential resistance to structurally diverse ALK inhibitors. Clin. Cancer Res..

[26] Hrustanovic G (2015). RAS-MAPK dependence underlies a rational polytherapy strategy in EML4-ALK–positive lung cancer. Nat. Med..

[27] Crystal AS (2014). Patient-derived models of acquired resistance can identify effective drug combinations for cancer. Science.

[28] Lederer S, Dijkstra TMH, Heskes T (2018). Additive Dose Response Models: Explicit Formulation and the Loewe Additivity Consistency Condition. Front Pharmacol.

[29] Yeh TC (2007). Biological characterization of ARRY-142886 (AZD6244), a potent, highly selective mitogen-activated protein kinase kinase 1/2 inhibitor. Clin Cancer Res.

[30] Davies BR (2007). AZD6244 (ARRY-142886), a potent inhibitor of mitogen-activated protein kinase/extracellular signal-regulated kinase kinase 1/2 kinases: mechanism of action *in vivo*, pharmacokinetic/pharmacodynamic relationship, and potential for combination in preclinical. Mol. Cancer Ther..

[31] Hainsworth JD (2010). A phase II, open-label, randomized study to assess the efficacy and safety of AZD6244 (ARRY-142886) versus pemetrexed in patients with non-small cell lung cancer who have failed one or two prior chemotherapeutic regimens. J. Thorac. Oncol..

[32] Garon EB (2010). Identification of Common Predictive Markers of *In vitro* Response to the Mek Inhibitor Selumetinib (AZD6244; ARRY-142886) in Human Breast Cancer and Non-Small Cell Lung Cancer Cell Lines. Mol. Cancer Ther..

[33] Sabir, S. R., Yeoh, S., Jackson, G. & Bayliss, R. EML4-ALK Variants: Biological and Molecular Properties, and the Implications for Patients. *Cancers (Basel)* **9**, 10.3390/cancers9090118 (2017). 10.3390/cancers9090118 PMC5615333 28872581

[34] Cha YJ, Kim HR, Shim HS (2016). Clinical outcomes in ALK-rearranged lung adenocarcinomas according to ALK fusion variants. J. Transl. Med..

[35] Wilson C, Nimick M, Nehoff H, Ashton JC (2017). ALK and IGF-1R as independent targets in crizotinib resistant lung cancer. Sci. Rep..

[36] Vichai V, Kirtikara K (2006). Sulforhodamine B colorimetric assay for cytotoxicity screening. Nat. Proto.c.

[37] Chou T-C, Talalay P (1983). Analysis of combined drug effects: a new look at a very old problem. Trends in Pharmacol. Sci..

[38] Goldoni M, Johansson C (2007). A mathematical approach to study combined effects of toxicants *in vitro*: evaluation of the Bliss independence criterion and the Loewe additivity model. Toxicol. In Vitro.

[39] Holt SV (2012). The MEK1/2 inhibitor, selumetinib (AZD6244; ARRY-142886), enhances anti-tumour efficacy when combined with conventional chemotherapeutic agents in human tumour xenograft models. Br. J. Cancer.

[40] Camidge DR, Doebele RC (2012). Treating ALK-positive lung cancer—early successes and future challenges. Nat.Rev. Clin.Oncol..

[41] Ziogas DC (2018). Treating ALK-positive non-small cell lung cancer. Ann. Transl. Med..

[42] Sale MJ, Cook SJ (2013). The BH3 mimetic ABT-263 synergizes with the MEK1/2 inhibitor selumetinib/AZD6244 to promote BIM-dependent tumour cell death and inhibit acquired resistance. Biochem. J..

[43] Christensen JG (2007). Cytoreductive antitumor activity of PF-2341066, a novel inhibitor of anaplastic lymphoma kinase and c-Met, in experimental models of anaplastic large-cell lymphoma. Mol. Cancer Therap..

[44] Zhou Y (2016). Selumetinib suppresses cell proliferation, migration and trigger apoptosis, G1 arrest in triple-negative breast cancer cells. BMC Cancer.

[45] Bhatt KV (2005). Adhesion control of cyclin D1 and p27Kip1 levels is deregulated in melanoma cells through BRAF-MEK-ERK signaling. Oncogene.

[46] Tanizaki J (2011). MET tyrosine kinase inhibitor crizotinib (PF-02341066) shows differential antitumor effects in non-small cell lung cancer according to MET alterations. J. Thorac.Oncol..

[47] Bhalla S (2011). The novel anti-MEK small molecule AZD6244 induces BIM-dependent and AKT-independent apoptosis in diffuse large B-cell lymphoma. Blood.

[48] Ley R, Balmanno K, Hadfield K, Weston C, Cook SJ (2003). Activation of the ERK1/2 signaling pathway promotes phosphorylation and proteasome-dependent degradation of the BH3-only protein, Bim. J. Biol. Chem..

[49] Hamedani FS (2014). Crizotinib (PF-2341066) induces apoptosis due to downregulation of pSTAT3 and BCL-2 family proteins in NPM-ALK(+) anaplastic large cell lymphoma. Leuk. Res..

[50] Zheng X, He K, Zhang L, Yu J (2013). Crizotinib induces PUMA-dependent apoptosis in colon cancer cells. Mol. Cancer Ther..

