# Supplementary material for: Mechanisms of suppression of cell growth by dual inhibition of ALK and MEK in ALK-positive non-small cell lung cancer
Source: Sci Rep. 2019 Dec 11;9:18842. doi: 10.1038/s41598-019-55376-4 (PMC6906283; doi:10.1038/s41598-019-55376-4)
Supplement: Supplementary file 1 — Supplementary material [file 41598_2019_55376_MOESM1_ESM.pdf]

Supplementary Materials

Mechanisms of suppression of cell growth by dual inhibition of ALK and MEK in ALK-positive non-small cell lung cancer

Shrestha, N., Nimick, M., Dass, P., Rosengren, R.J., Ashton, J.C[1].

Department of Pharmacology & Toxicology, School of Biomedical Sciences, University of Otago, Dunedin, New Zealand.

1. Corresponding author
   +6434793040
   john.ashton@otago.ac.nz

Running Head: Dual inhibition of ALK and MEK in ALK-positive lung cancer

Keywords: Crizotinib; Selumetinib; ALK-positive lung cancer; Combination treatment; Synergy

Table S1: ALK kinase domain sequences of H3122 and CR-H3122 cell lines. The kinase domain of ALK was amplified from H3122 and CR-H3122 cDNA using PCR primers (Table S2). After gel purification the PCR products were sequenced using Sanger sequencing. The sequences of exons 22-25 (NM_004304), corresponding to the kinase domain are indicated in bold.

Cell line	ALK domain sequence (forward strand, 5’ to 3’)
H3122	CCTCATTCGGGGTCTGGGCCATGGCGCCTTTGGGGAGGTGTATGA<br>AGGCCAGGTGTCCGGAATGCCCAACGACCCAAGCCCCCTGCAAG<br>TGGCTGTGAAGACGCTGCCTGAAGTGTGCTCTGAACAGGACGAA<br>CTGGATTTCCTCATGGAAGCCCTGATCATCAGC**AAATTCAACCA**<br>**CCAGAACATTGTTCGCTGCATTGGGGTGAGCCTGCAATCCCT**<br>**GCCCCGGTTCATCCTGCTGGAGCTCATGGCGGGGGGAGACC**<br>**TCAAGTCCTTCCTCCGAGAGACCCGCCCTCGCCCGAGCCAGC**<br>**CCTCCTCCCTGGCCATGCTGGACCTTCTGCACGTGGCTCGGG**<br>**ACATTGCCTGTGGCTGTCAGTATTTGGAGGAAAACCACTTCA**<br>**TCCACCGAGACATTGCTGCCAGAAACTGCCTCTTG**ACCTGTCC<br>AGGCCCTGGAAGAGTGGCCAAGATTGGAGACTTCGGGATGGCCC<br>GAGACATCTACAGGGCGAGCTACTATAGAAAGG
CR-H3122	CTCATTCGGGGTCTGGGCCATGGCGCCTTTGGGGAGGTGTATGAA<br>GGCCAGGTGTCCGGAATGCCCAACGACCCAAGCCCCCTGCAAGT<br>GGCTGTGAAGACGCTGCCTGAAGTGTGCTCTGAACAGGACGAAC<br>TGGATTTCCTCATGGAAGCCCTGATCATCAGC**AAATTCAACCAC**<br>**CAGAACATTGTTCGCTGCATTGGGGTGAGCCTGCAATCCCTG**<br>**CCCCGGTTCATCCTGCTGGAGCTCATGGCGGGGGGAGACCTC**<br>**AAGTCCTTCCTCCGAGAGACCCGCCCTCGCCCGAGCCAGCCC**<br>**TCCTCCCTGGCCATGCTGGACCTTCTGCACGTGGCTCGGGAC**<br>**ATTGCCTGTGGCTGTCAGTATTTGGAGGAAAACCACTTCATC**<br>**CACCGAGACATTGCTGCCAGAAACTGCCTCTTG**ACCTGTCCAG<br>GCCCTGGAAGAGTGGCCAAGATTGGAGACTTCGGGATGGCCCGA<br>GACATCTACAGGGCGAGCTACTATAGAAAGG

Table S2: Primer sequences used for amplification and sequencing of ALK kinase domain.

**Primer**	**Sequence (5’ to 3’)**
ALK_3_F (forward)	CCATCAGTGACCTGAAGGAGG
ALK_3_R (reverse)	TGGCACAGCCTCCCTTTCTAC

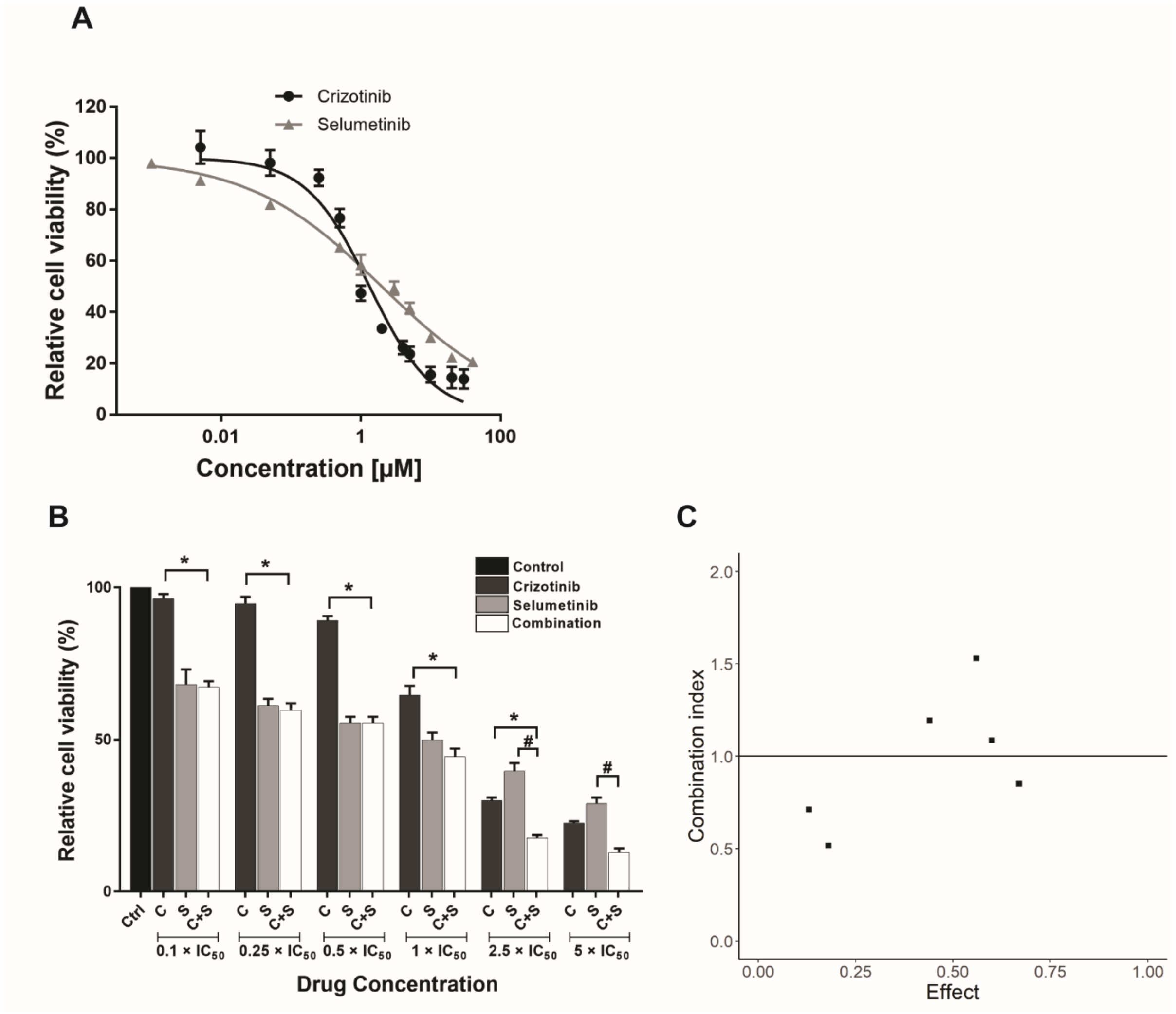

Figure S1 : Effect of crizotinib, selumetinib and their combination in ALK-negative non-small cell lung cancer (A549) cells. (A) Cytotoxicity of ALK inhibitor; crizotinib and MEK inhibitor; selumetinib, data points are means and error bars are SEM. (B) Cytotoxicity of a combination of crizotinib and selumetinib. Bar denotes; ■ DMSO control, ■ crizotinib, ■ selumetinib and □ combination of both. Errors bars are SEM. (C) Combination index plot for drug combination of crizotinib and selumetinib. The horizontal line represents additivity, below the line represents synergy and above the line represents inhibition. All data represent three independent experiments in triplicate. *p < 0.05 for crizotinib vs. combination and #p < 0.05 for selumetinib vs. combination.

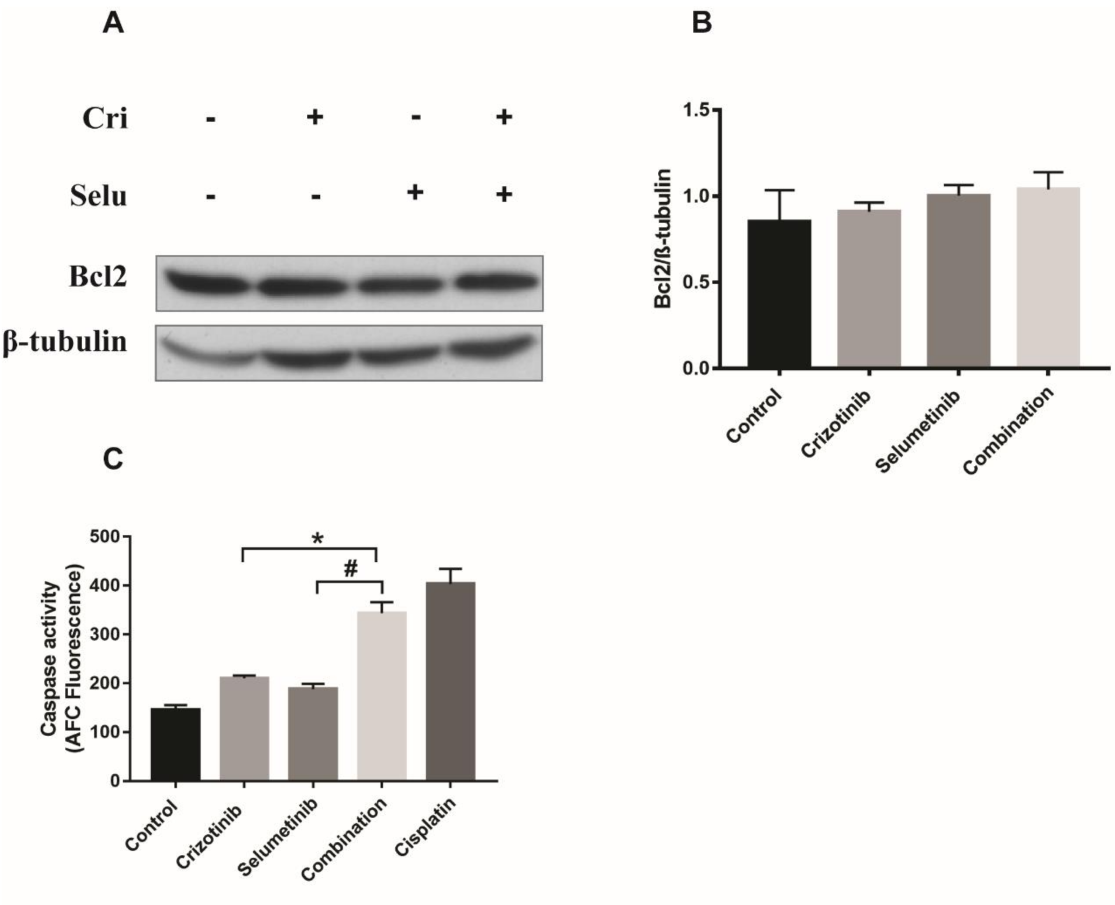

Figure S2: Effect of crizotinib, selumetinib and their combination on Bcl2 and caspase activity in H3122 cells. (A) Representative Western blots of anti-apoptotic marker; Bcl2. (B) Densitometry of Bcl2. (C) Caspase activity after treatment with indicated concentration of crizotinib, selumetinib, their combination and cisplatin (positive control) for 48 h. All data are presented as mean ± SEM. Three independent experiment were performed. *p < 0.05 for crizotinib vs. combination and #p < 0.05 for selumetinib vs. combination.

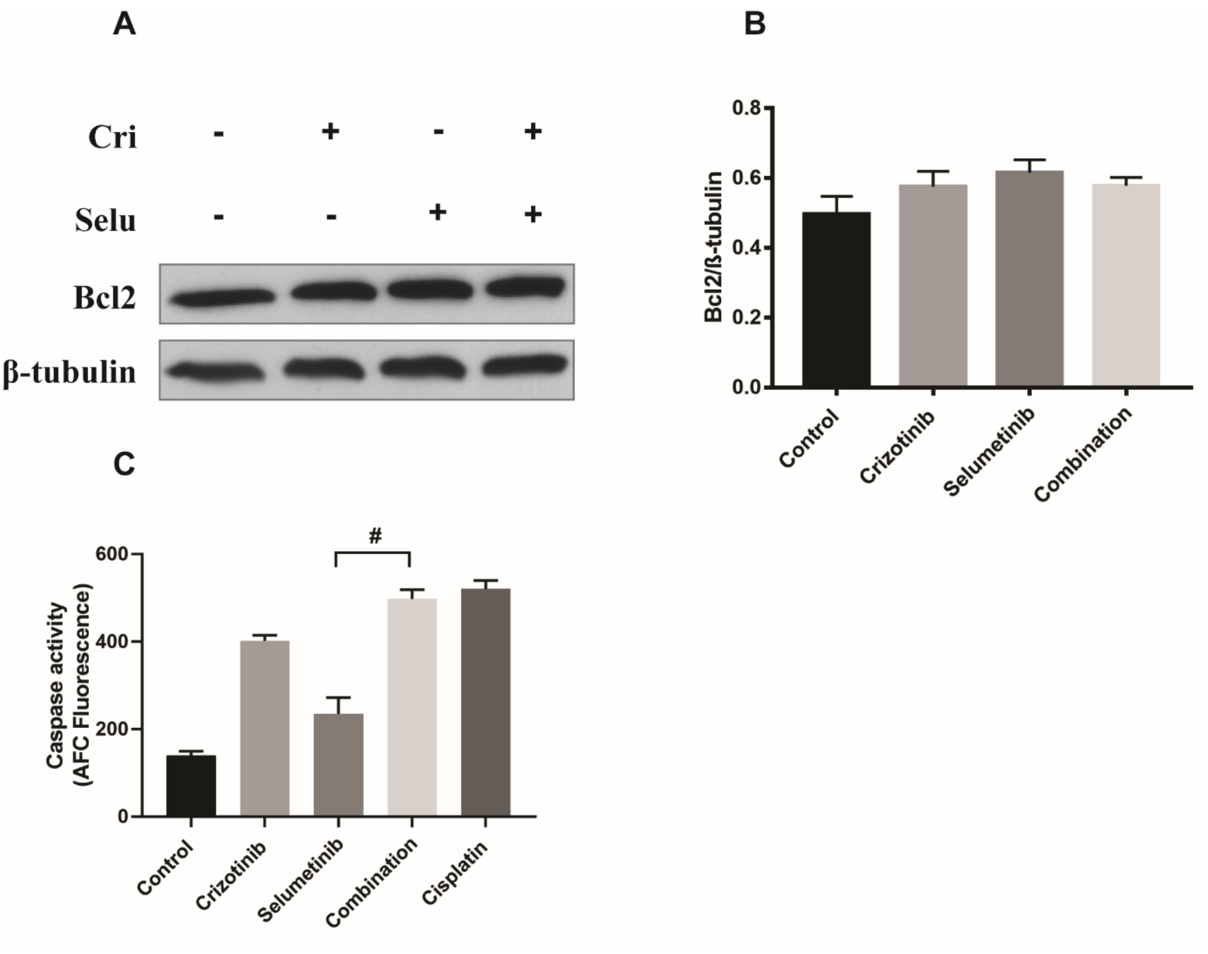

Figure S3 : Effect of crizotinib, selumetinib and their combination on Bcl2 and Caspase activity in CRH3122 cells. (A) Representative Western blots of anti-apoptotic marker; Bcl2. (B) Densitometry of Bcl2. (C) Caspase activity after treatment with indicated concentration of crizotinib, selumetinib, their combination and cisplatin (positive control) for 24 h. All data are presented as mean ± SEM. Three independent experiment were performed. *p < 0.05 for crizotinib vs. combination and #p < 0.05 for selumetinib vs. combination.

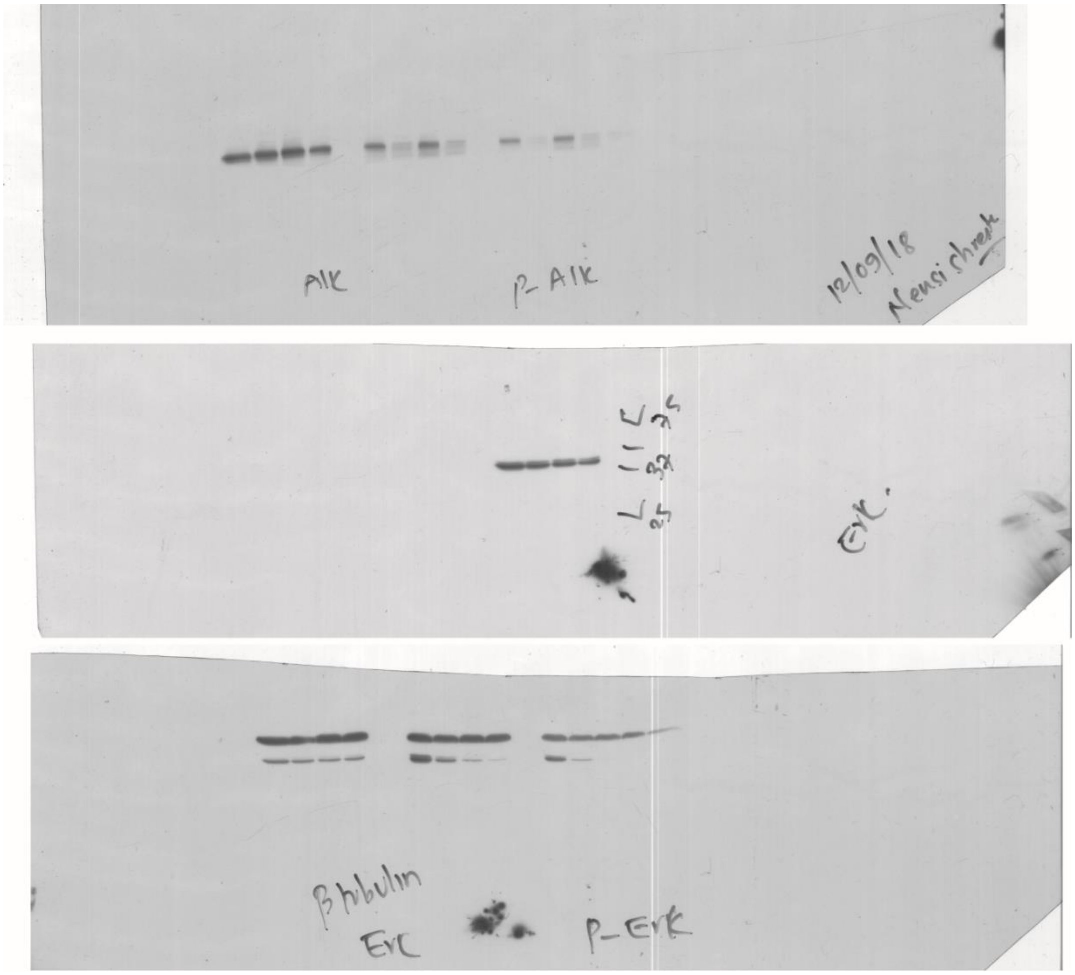

Figure S4 : Raw image of Western blots for ALK, pALK, ERK, pERK and β-tubulin in H3122 cells used in Figure 2A.

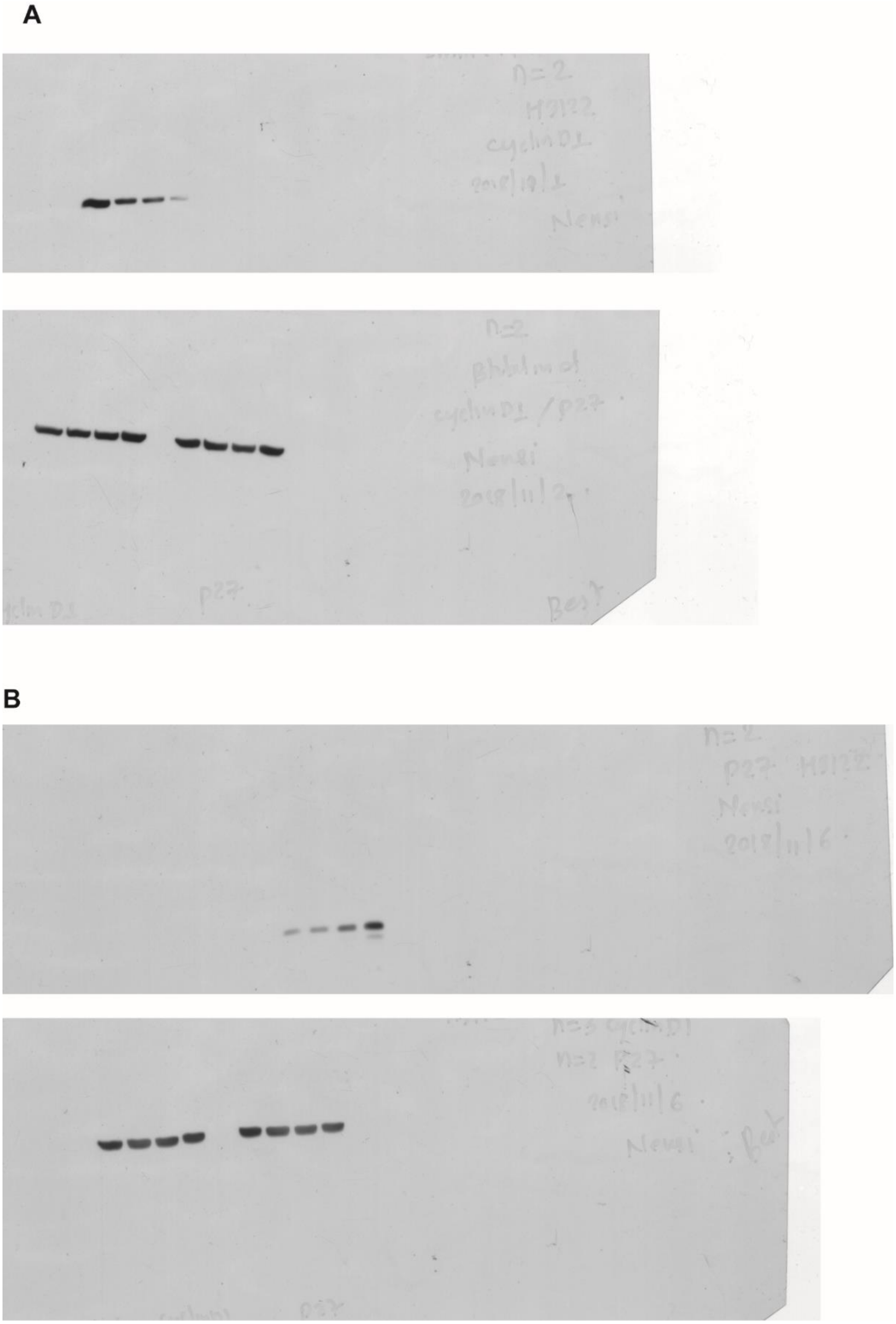

Figure S5: Raw image of Western blots for CyclinD1, p27 and β-tubulin in H3122 cells used in Figure 2A.

A

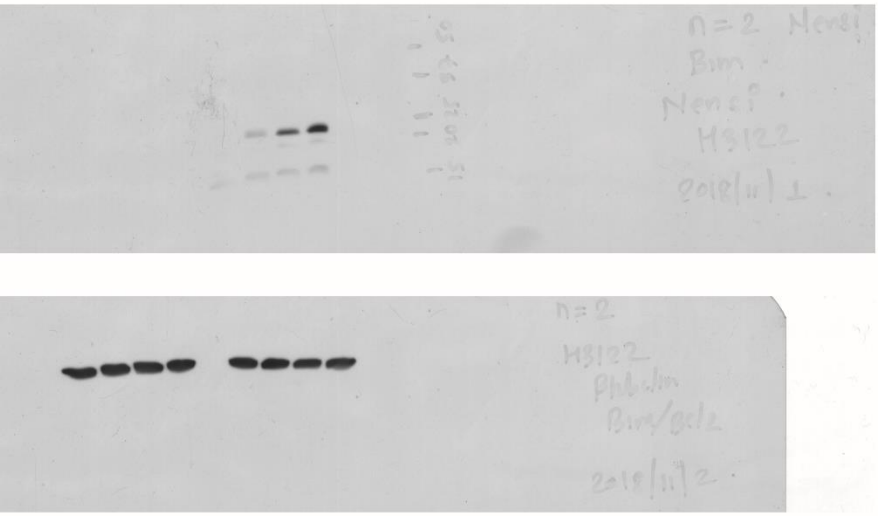

B

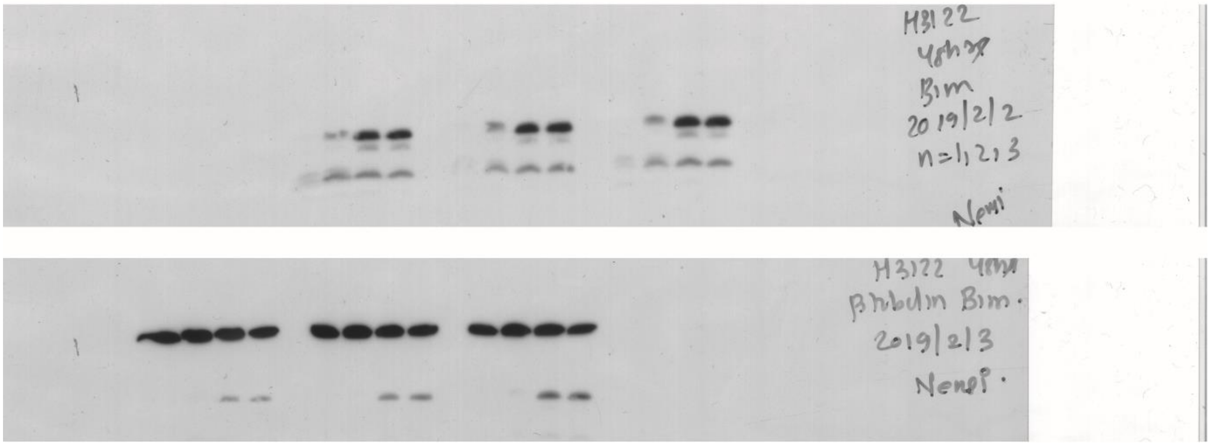

Figure S6: Raw image of Western blots for Bim after 24 and 48 h of drug treatment in H3122 cells used in Figure 3B.

A

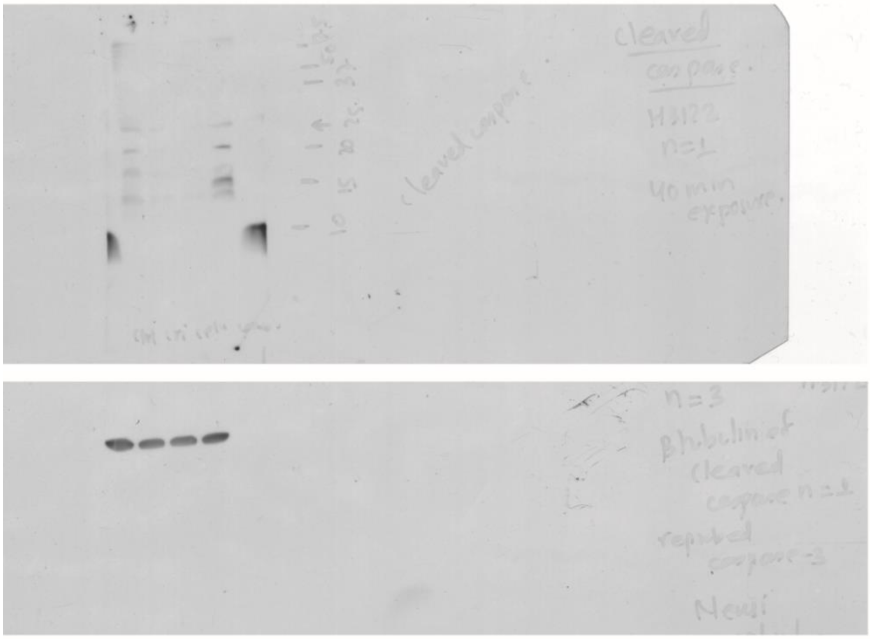

B

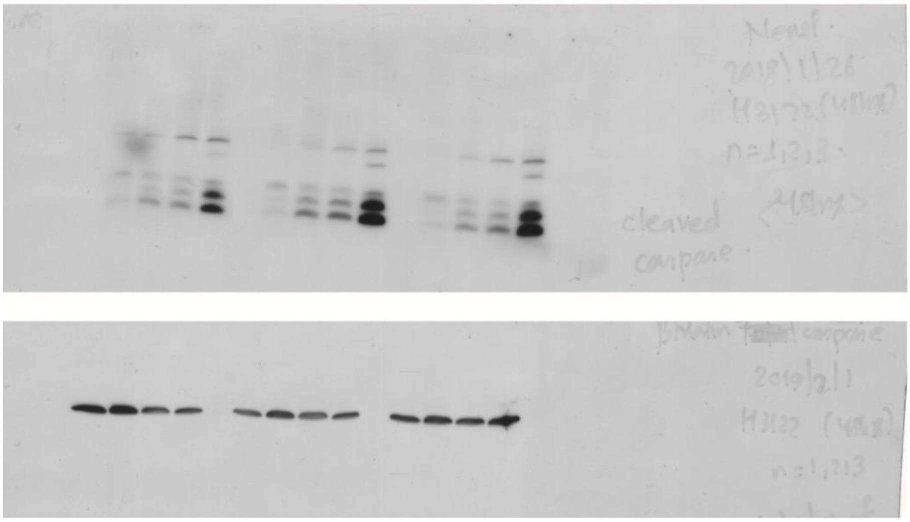

Figure S7 : Raw image of Western blots for cleaved Caspase after 24 and 48 h of drug treatment in H3122 cells used in Figure 3B.

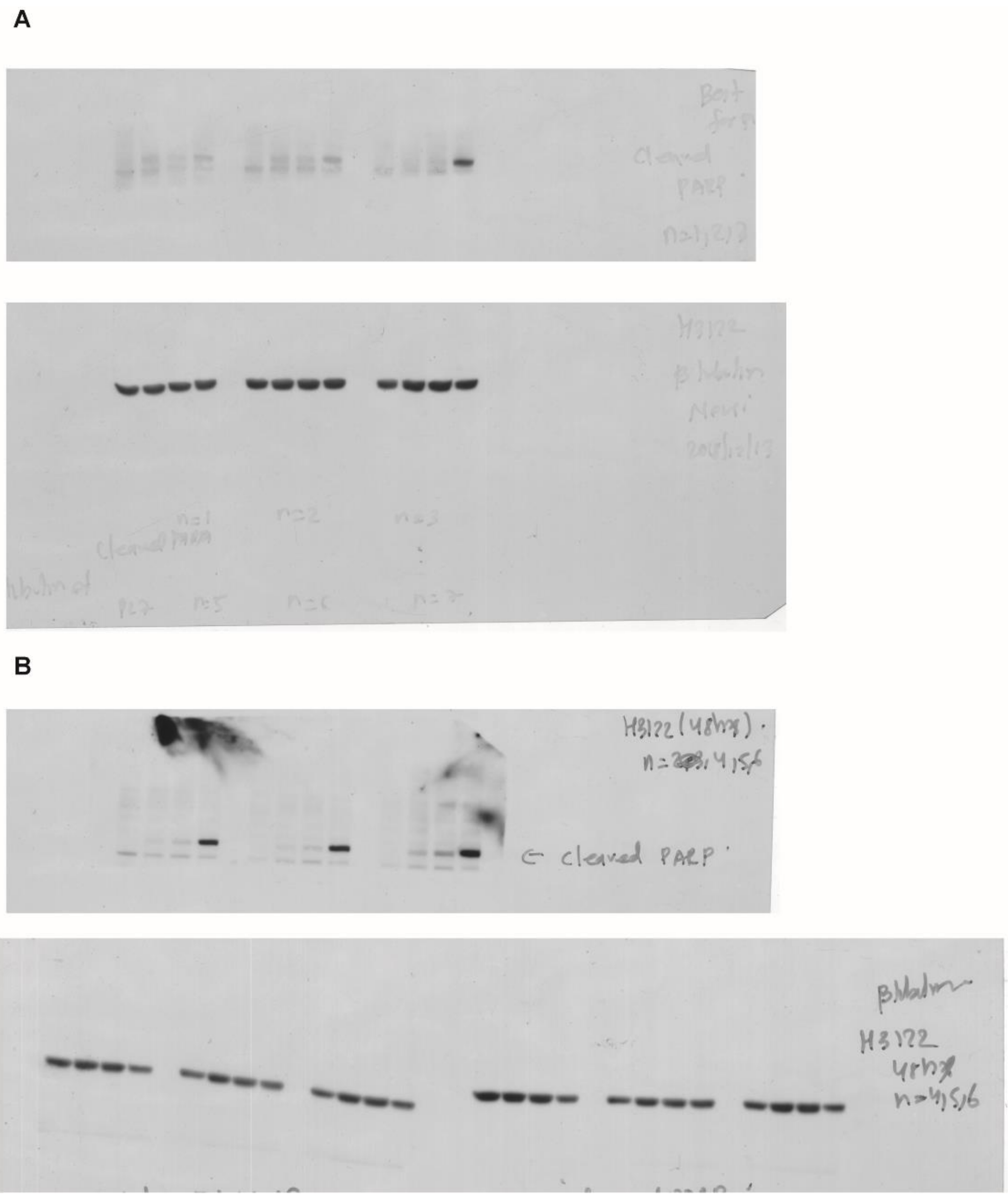

Figure S8 : Raw image of Western blots for cleaved PARP after 24 and 48 h of drug treatment in H3122 cells used in Figure 3B.

A

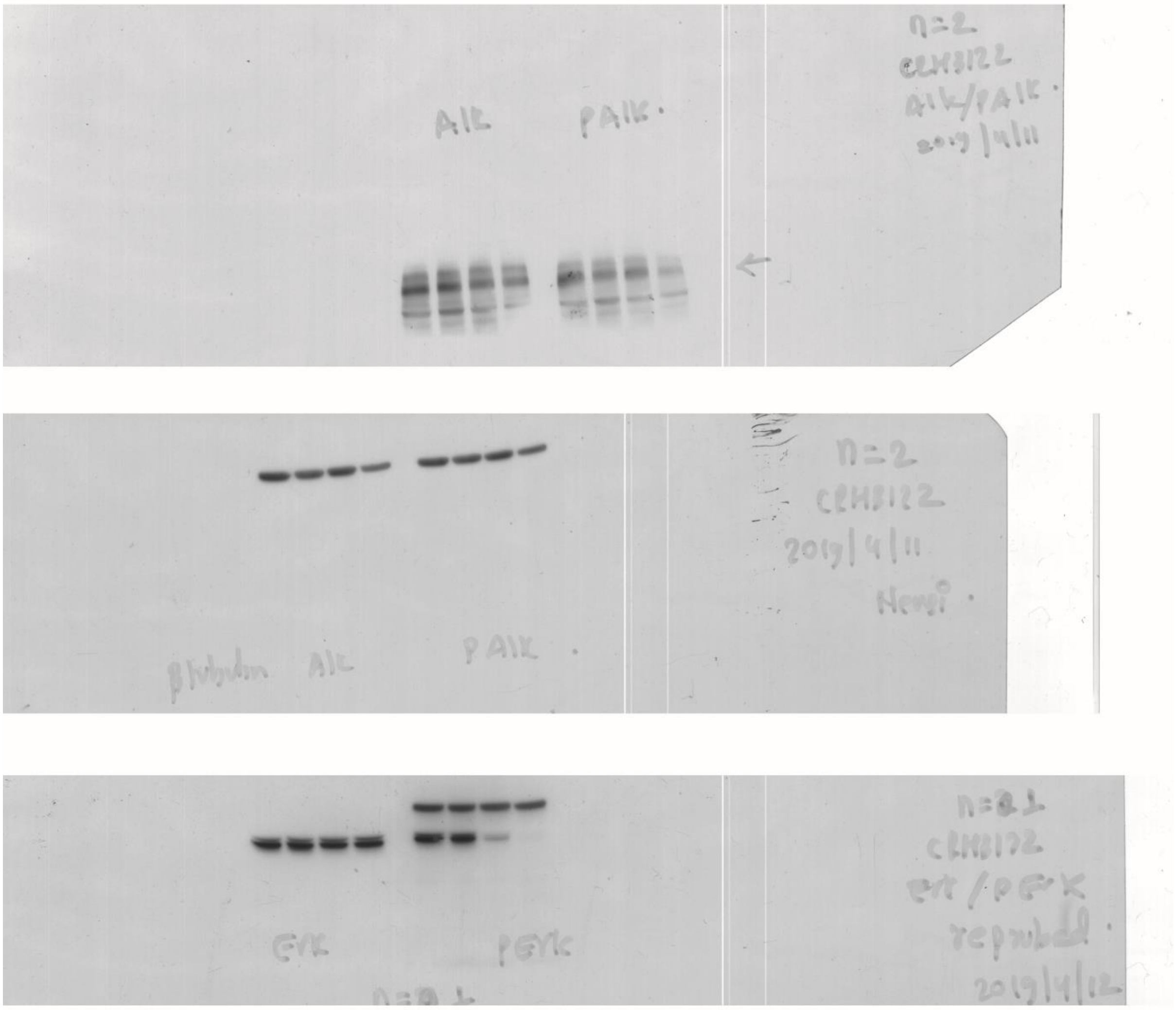

Figure S4 : Raw image of Western blots for ALK, pALK, ERK, pERK and β-tubulin in CRH3122 cells used in Figure 5A.

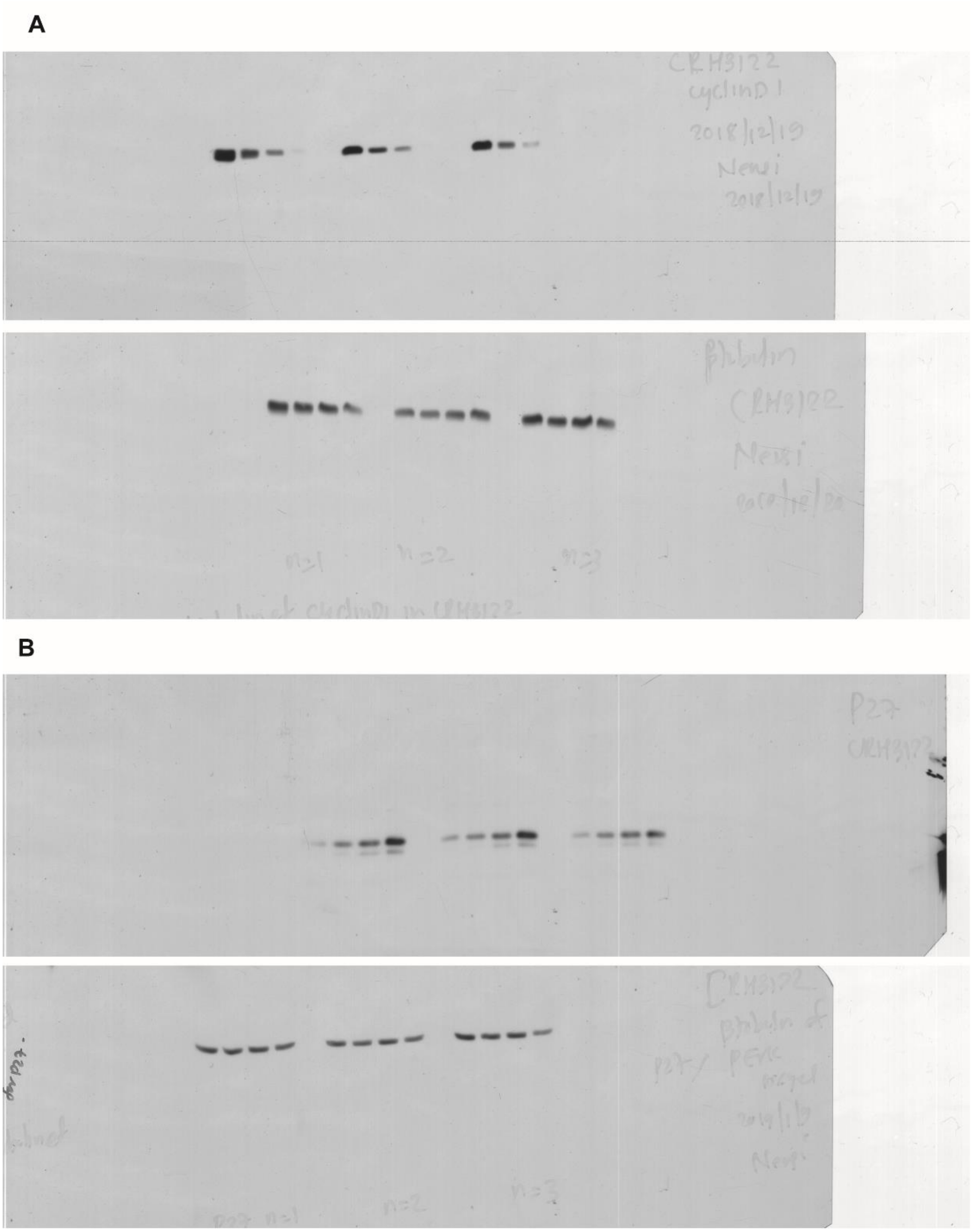

Figure S10: Raw image of Western blots for CyclinD1, p27 and β-tubulin in CRH3122 cells used in Figure 5A.

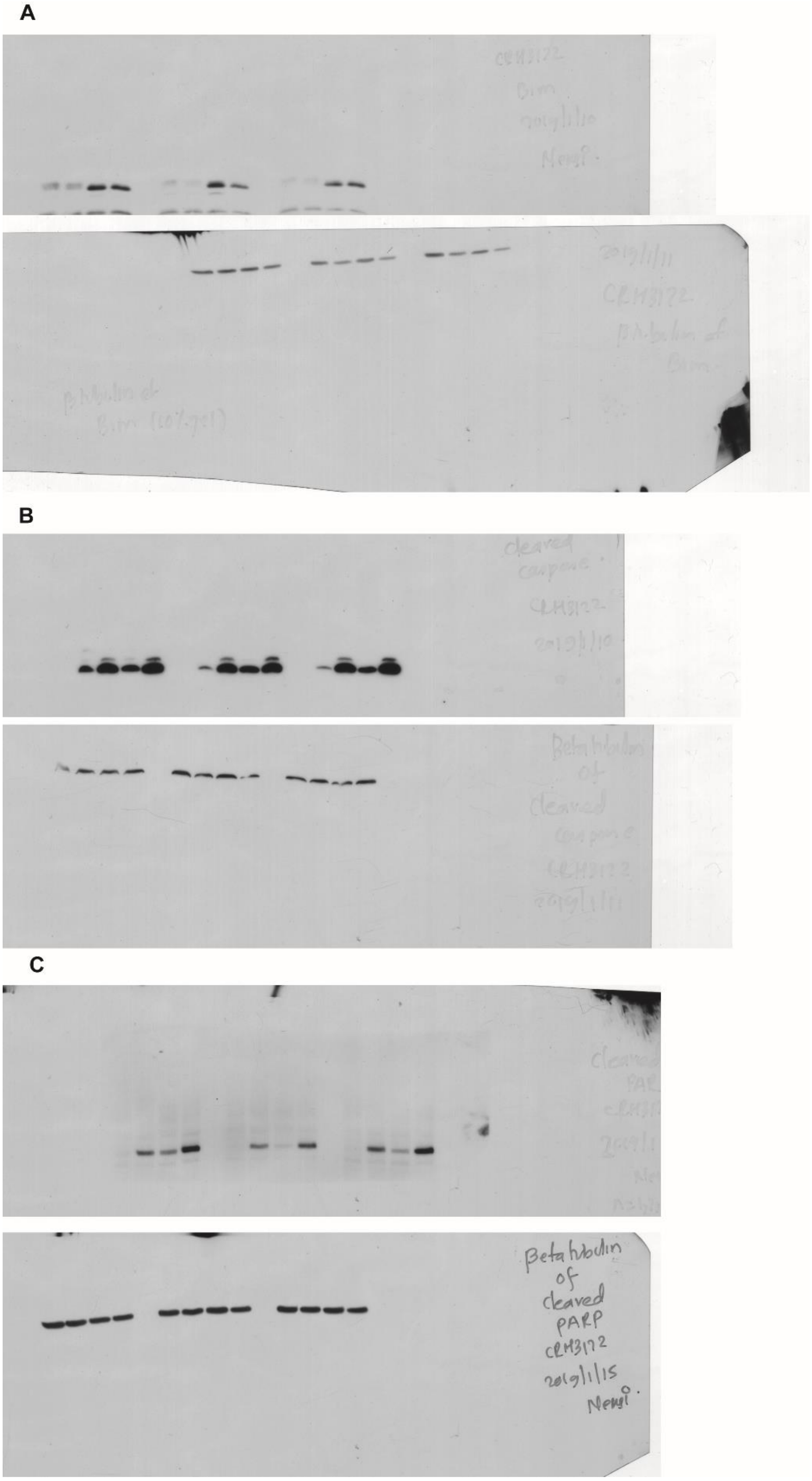

Figure 11 : Raw image of Western blots for Bim, cleaved Caspase, cleaved PARP and β-tubulin after 24 h of drug treatment in CRH3122 cells used in Figure 5A.
